# “Porous and Yet Dense” Electrodes for High‐Volumetric‐Performance Electrochemical Capacitors: Principles, Advances, and Challenges

**DOI:** 10.1002/advs.202103953

**Published:** 2021-11-18

**Authors:** Zhenghui Pan, Jie Yang, Junhua Kong, Xian Jun Loh, John Wang, Zhaolin Liu

**Affiliations:** ^1^ Department of Materials Science and Engineering National University of Singapore Singapore 117574 Singapore; ^2^ Department of Electrical and Computer Engineering National University of Singapore Singapore 117583 Singapore; ^3^ Institute of Materials Research and Engineering (IMRE) A*STAR (Agency for Science, Technology and Research) 2 Fusionopolis Way Singapore 138634 Singapore

**Keywords:** electrochemical capacitors, high volumetric performance, portable and wearable electronics, “porous and yet dense” electrodes

## Abstract

With the ever‐rapid miniaturization of portable, wearable electronics and Internet of Things, the volumetric performance is becoming a much more pertinent figure‐of‐merit than the conventionally used gravimetric parameters to evaluate the charge‐storage capacity of electrochemical capacitors (ECs). Thus, it is essential to design the ECs that can store as much energy as possible within a limited space. As the most critical component in ECs, “porous and yet dense” electrodes with large ion‐accessible surface area and optimal packing density are crucial to realize desired high volumetric performance, which have demonstrated to be rather challenging. In this review, the principles and fundamentals of ECs are first observed, focusing on the key understandings of the different charge storage mechanisms in porous electrodes. The recent and latest advances in high‐volumetric‐performance ECs, developed by the rational design and fabrication of “porous and yet dense” electrodes are then examined. Particular emphasis of discussions then concentrates on the key factors impacting the volumetric performance of porous carbon‐based electrodes. Finally, the currently faced challenges, further perspectives and opportunities on those purposely engineered porous electrodes for high‐volumetric‐performance EC are presented, aiming at providing a set of guidelines for further design of the next‐generation energy storage devices.

## Graphical abstract

“Porous and yet dense” electrodes with optimum structures that balance the level of porosity and packing density are crucial to realize high‐volumetric‐performance electrochemical capacitors. This review presents design principles, recent achievements, and further perspectives on “porous and yet dense” electrodes, aiming at providing a set of guidelines for development of the nextgeneration advanced electrochemical capacitors.

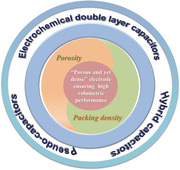

## 1 Introduction

**Figure 1:**
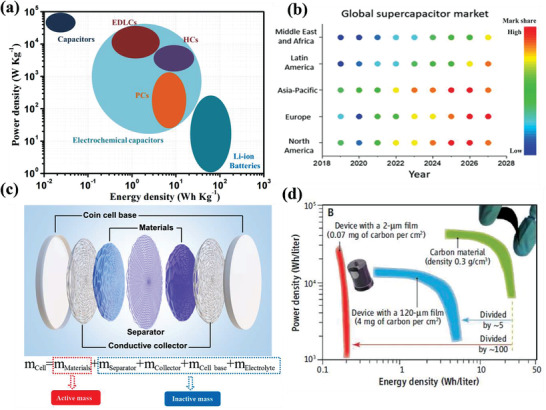
a) Ragone plots of traditional capacitors, electrochemical capacitors (including EDLCs, PCs, and HCs), and Li‐ion batteries. b) Global ECs market share maps according to regions (*y*‐axis) and years (*x*‐axis). Reproduced with permission.^[^ ^16^ ^]^ Copyright 2021, Royal Society of Chemistry. c) A schematic illustration of the construction of the commercial coin cell. d) The Ragone plots for the same ECs are on a volumetric basis. Reproduced with permission.^[^ ^17^ ^]^ Copyright 2011, American Association for the Advancement of Science (AAAS).

Exploration for renewable, sustainable, and environment‐friendly energy sources (e.g., solar, wind, and ocean) is among the key advances as well as challenges in the 21st century, in efforts to address issues in relation to both the rapid depletion of fossil fuels and worsening climate‐changing issues.^[^ ^1^, ^2^ ^]^ In the coming 10 to 15 years, human civilization will witness the great electrification of the entire transport systems, where electric vehicles will replace the traditional combustion‐powdered transports. Reliable energy storage technologies/systems are playing an essential role to ensure the energy and power structure revolutions. Lithium‐ion batteries (LIBs) have been largely dominating the current market, since they were introduced in 1990 by Sony. On the one hand, there is no doubt that LIBs can fulfil a large part of the basic energy and power requirement. On the other hand, they are known to suffer from a somewhat low power density, limited cycle life, intrinsically unsafe due to the use of organic electrolytes, making them difficult to satisfy the high‐power delivery/uptake and long‐cycle stability applications.^[^ ^3^, ^4^ ^]^ Electrochemical capacitors (ECs), also called supercapacitors, fill in the large gap of electrochemical energy storage systems/applications requiring fast‐charging capability, high power density, and extra‐long cycling life (**Figure** **1**a).^[^ ^5^, ^6^, ^7^, ^8^, ^9^, ^10^, ^11^, ^12^, ^13^, ^14^, ^15^ ^]^ Over the past three decades, ECs have been extensively developed to complement or even replace part of the functions played by LIBs in several practical fields, where, e.g., a high power density of above 10 kW kg^−1^ is required for a short time period (a few seconds). There has been a steady and continuing rise of the global EC market over recent years (Figure 1b).^[^ ^16^ ^]^ However, an imperative need is to improve their low energy densities (less than 10 W h kg^−1^), preferably close to those of LIBs (more than 150 W h kg^−1^), which will significantly extend their application roadmap in the on‐going ever‐growing energy requirement.^[^ ^5^, ^17^, ^18^ ^]^

ECs can be generally classified into three categories, based on the charge storage mechanisms and types of active materials used: electrochemical double layer capacitors (EDLC), pseudocapacitors (PC), and hybrid capacitors (HC).^[^ ^19^, ^20^, ^21^, ^22^, ^23^, ^24^ ^]^ The EDLCs store energy by physically charging the electrical double layers through highly reversible ion adsorption at the interface between the electrolyte and electrode, where carbon‐based materials (e.g., activated carbon (AC), carbon nanotube (CNT), and graphene) are the dominant material choices.^[^ ^8^, ^10^, ^25^, ^26^, ^27^, ^28^ ^]^ Thus, an appropriate porous electrode made of carbon‐based materials with high ion‐accessible surface area, good electrical conductivity, and high chemical stability in electrolytes is crucial to realize a class of high‐performance EDLCs.^[^ ^7^, ^11^, ^12^, ^14^, ^28^ ^]^ For PCs, because they store the charges via chemically fast and reversible Faradaic reactions at the surface/near‐surface of the transition metal oxides or conductive polymers‐based electrodes (e.g., RuO_2_, MnO_2_, polypyrrole, and polyaniline), advanced pseudocapacitive electrodes shall also possess an appropriate porous structure, which not only benefits the electrolyte penetrating into active materials, but also largely speeds up the ion transport across the entire surface.^[^ ^19^, ^20^, ^21^, ^22^, ^23^, ^25^, ^26^, ^27^, ^29^ ^]^ Although the HCs offer an opportunity to take the advantage of the merits of both ECs and batteries,^[^ ^19^, ^24^, ^30^ ^]^ the wide charge/capacity gap remaining between capacitive or pseudocapacitive electrode (low capacity) and battery‐type electrode (high capacity) is the main barrier for realizing an overall high‐performance HC (particularly the desired energy and power densities, and long cycle‐life). It would be necessary to develop advanced electrodes based on porous carbon or pseudocapacitive materials showing higher capacity (approaching or comparable to the value of battery‐type electrode) to address this limitation.

More recently, tremendous research efforts have been devoted to improving the energy density of ECs by tuning the highly porous electrodes, in which the surface conditions and pore structures are designed to precisely couple with the fluid electrolytes, while at the same time optimizing the interfaces involved.^[^ ^7^, ^8^, ^11^, ^12^, ^28^, ^31^ ^]^ Broadening the working voltage window of ECs is another effective approach to increase their energy densities, where, e.g., it could be realized by employing an appropriate electrolyte chosen from ionic liquids (ILs) and organic electrolytes that allow for a large voltage window (up to 2.5–4.0 V) and the development of asymmetric ECs (≈1.8–2.6 V); similarly, an appropriately porous structure is also needed to match with these large size of electrolyte ions.^[^ ^32^, ^33^ ^]^

There have been a large number of research works that focus on raising the gravimetric capacitive performance by tuning the porous electrodes, where limited consideration is given the volumetric performance.^[^ ^34^, ^35^, ^36^, ^37^ ^]^ However, a high surface area with low packing density will unfortunately result in abundant void spaces/pores in the electrodes that could be flooded by the electrolytes, thereby adding on the weight of device without contributing to extra capacitance. In fact, it reduces the volumetric energy density significantly. Taking a carbon aerogel as an example, it possesses a very high level of porosity at 90% and high surface area (per mass), but its volumetric energy density is only 20% that of a typical carbon electrode with ≈50% porosity.^[^ ^38^ ^]^ Indeed, as Gogotsi and Simon have pointed out, the energy and power densities per weight of the active material on a Ragone plot may not effectively provide an actual picture of the performance that a device could reach (Figure 1c).^[^ ^17^ ^]^ In contrast, the volumetric performance is much more relevant and a more valuable merit than the traditionally used gravimetric one to assess the charge‐storage capacity of ECs, especially for the situations with space limitations, which is so by considering the rapid development and miniaturization of portable and wearable electronics.^[^ ^39^ ^]^ For example, an extremely high gravimetric energy density of 85 Wh kg^−1^ can be achieved for selected porous graphene electrodes with low packing density (0.3 g cm^−3^). However the volumetric energy densities are only in the range of ≈25.5 Wh L^−1^ for the same electrodes and ≈5.0 Wh L^−1^ for the device (Figure 1d), which is far less than those of the conventional lead acid batteries (50–90 Wh L^−1^) and LIBs (250–850 Wh L^−1^).^[^ ^16^, ^17^ ^]^ Therefore, it would be highly desirable to design and develop a class of advanced EC electrodes with optimum structures that effectively balance the level of porosity (or ion‐accessible surface area) and the packing density, which shall simultaneously deliver high gravimetric and volumetric energy densities, without sacrificing the power density and cycle life to fulfil the requirement for the rapidly changing applications.

Over the past few years, there has been a steady progress in improving the volumetric performance of EDLC, PC, and HC, by engineering “porous and yet dense” electrodes, especially those carbon‐based, and there are a few reviews published lately in the on‐going hot topic area.^[^ ^7^, ^8^, ^10^, ^11^, ^14^, ^15^ ^]^ For example, Yang et al. have reviewed advances on the dense and yet porous electrode with superior volumetric performance from a pore‐engineering perspective.^[^ ^8^ ^]^ Simon et al. briefly summarized the then up‐to‐date advances in nanoporous carbon‐based electrodes.^[^ ^15^ ^]^ However, there is a timely need for an updated, critical, and comprehensive overview covering all key aspects of the porous electrodes for high‐volumetric‐performance EC, from the viewpoint of design principles, key advances, and future challenges. In this review, we will briefly visit the importance of volumetric performance as a key relevant parameter, and discuss the principles and fundamentals of ECs. The recent advances made through the rational design and development of various “porous and yet dense” electrodes with high volumetric performance are examined. In particular, we present a discussion on the key factors that impact the volumetric performance of carbon‐based electrodes, which are the dominant choices for ECs. The currently faced challenges and future opportunities on these “porous and yet dense” electrode for high‐volumetric‐performance EC are outlined, aiming at sparking new ideas and endeavors in bringing ECs into large scale and widening applications.

## 2 Principles and Fundamentals of ECs

### 2.1 Energy Storage Mechanisms

**Figure 2:**
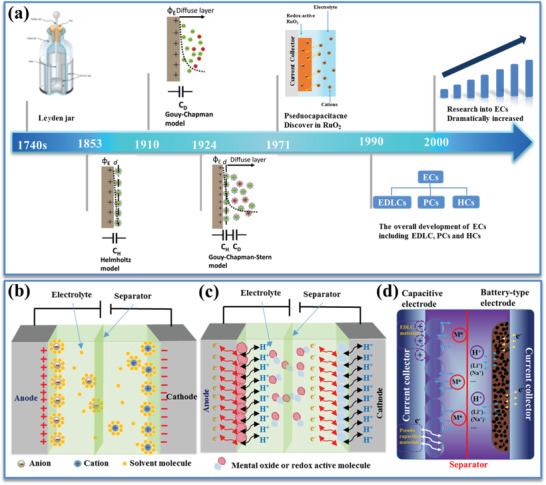
a) Historic timeline in development of ECs. Reproduced with permission.^[^ ^15^ ^]^ Copyright 2020, Royal Society of Chemistry. General energy storage mechanisms and device structure of three types of EC: b) EDLC, c) PC, and d) HC.

The long story of investigating charge‐storage mechanisms of ECs can be dated back to nearly 300 years ago, as illustrated in the timeline shown in **Figure** **2**a.^[^ ^21^, ^40^, ^41^, ^42^, ^43^, ^44^, ^45^, ^46^, ^47^, ^48^, ^49^ ^50]^ There has been tremendous progress made over the past three decades. Among the three types of EC, each of them possesses its own significant features.^[^ ^27^ ^]^ The first one is the EDLC, which is the most common device and employs the porous carbon‐based electrode materials. No electrochemical reactions take place on the electrode material during the charging/discharging process of EDLC, and the accumulated charges physically store at the electrolyte/electrode interface (Figure 2b). The second type is the PC making use of a fast and reversible Faradaic process at the surface/near‐surface reactions for charge storage, in which the pseudocapacitive electrode materials (e.g., transition metal oxides and electrically conducting polymers) are electrochemically active (Figure 2c). Thus, advanced pseudocapacitive electrodes shall also possess an appropriately porous structure, which not only benefits the electrolyte penetrating into active materials, but also largely speeds up the ion transport across the entire surface. The third one is the HC, based on a high‐rate capacitive electrode and a high‐capacity battery‐type electrode, which is designed with merits of both ECs and batteries (Figure 2d). Similarly, it is necessary to develop advanced porous carbon or pseudocapacitive electrodes showing higher capacity to address the limitation resulted from the wide charge/capacity gap between capacitive electrode and battery‐type electrode.

#### 2.1.1 EDLCs

**Figure 3:**
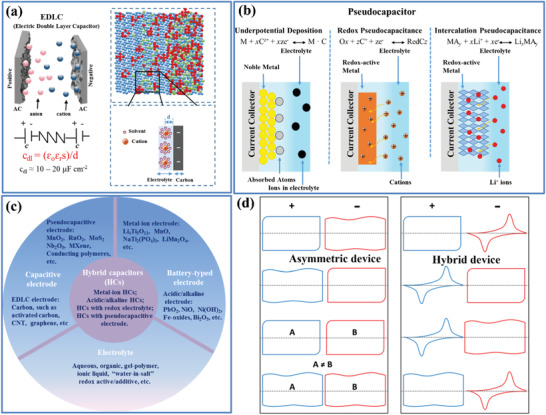
a) The upper left panel shows the schematic representation of an EDLC using porous carbon materials as the electrode materials. The upper right panel gives the simulation EDL cell consisting of a porous electrode filled with an IL (blue: carbon atoms, red: cation, and green: anion). The bottom left panel shows the equivalent circuit of an EDLC and the bottom right panel gives a schematic representation of EDL formation of a negative electrode. b) Three different types of pseudocapacitive electrodes: under‐potential deposition, redox PC, and ion intercalation PC. c) Various types of HC and their electrode and electrolyte materials. d) CV features for different EC configurations of asymmetric devices and hybrid devices (A and B are different materials). Reproduced with permission.^[^ ^20^ ^]^ Copyright 2019, Wiley‐VCH.

Essentially, an EDLC consists of two electrodes (positive and negative), an electrolyte, and a separator, which is similar to a typical battery. As shown in **Figure** **3**a, charges can be separated and stored at the interface between the electrolyte and the porous carbon nanoparticles. As the charge/discharge process, EDLC only involves the physically electrostatic charge adsorption without any Faradaic reactions, the formation and relaxation of the electric double layer can response immediately to potential changes within a very short time range of about 10^−8^ s, which is obviously smaller than that of the redox/Faradaic reactions in PCs (a time range of 10^−2^ to 10^−4^ s).^[^ ^14^, ^51^ ^]^

Considering the effective thickness of the electrical double layer is in the range of 0.5–1.0 nm, the electrical double‐layer capacitance for a carbon‐based EDLC is in a low value between 10 and 20 µF cm^−2^. For the past two decades, tremendous efforts had been focused on using porous carbon‐based materials ranging from commercial ACs, mesoporous carbon, carbon aerogels to carbon nanomaterials (e.g., CNTs, carbide‐derived carbons, and graphene) as the advanced electrodes for EDLCs, because of their large specific surface area (SSA), high open porosity to electrolyte ions, high electrical conductivity, and desirable chemical and thermal stabilities.^[^ ^52^ ^]^ For example, a variety of ACs with high SSAs in the range of 1000–3500 m^2^ g^−1^ have been successfully developed, which can lead to a double‐layer gravimetric capacitance of 300–550 F g^−1^ in aqueous electrolytes and 130–230 F g^−1^ in nonaqueous electrolytes.^[^ ^53^ ^]^ However, the volumetric capacitances of these AC‐based electrodes are only about 150–275 F cm^−3^ in aqueous electrolytes and 75–130 F cm^−3^ in nonaqueous electrolytes, because of their relatively low density (0.5 g cm^−3^). As a result, the volumetric energy density of commercially available EDLCs made of porous carbon‐based materials is about 5–8 Wh L^−1^, which is rather low.^[^ ^54^ ^]^

#### 2.1.2 PCs

In contrast to EDLCs, pseudocapacitive electrode materials chemically store charge via Faradaic processes that involve fast and reversible redox reactions at the surface/near‐surface of the active materials; but distinct from the reactions for the battery‐type materials, they do not experience phase transformations during the redox reactions. The widely known pseudocapacitive materials include transition metal oxide (e.g., RuO_2_, MnO_2_, Nb_2_O_5_, and MoO_3_), highly conducting polymers (e.g., polypyrrole and polyaniline) and recently emerging MXene.^[^ ^21^ ^]^ There are three common types of Faradaic mechanisms occurring at PC electrodes, which can lead to different electrochemical capacitive features as shown in Figure 3b: i) adsorption pseudocapacitance, which is well known for the adsorption of atoms (e.g., H and Pd) on catalytic noble metals (e.g., Pt and Au);^[^ ^55^ ^]^ ii) redox pseudocapacitance, where the redox reactions are described as electrochemical absorption of cations onto the surface/near‐surface of oxidized species (e.g., RuO_2_ or MnO_2_, and conducting polymers);^[^ ^56^, ^57^ ^]^ iii) intercalation pseudocapacitance, where ion intercalation/insertion into layered crystalline materials (e.g., Nb_2_O_5_ and LaMnO_3_) occurs without crystallographic phase change.^[^ ^58^ ^]^ When combining a capacitive electrode with a Faradaic electrode in an asymmetric EC, it is hard to differentiate whether the Faradaic electrode belongs to a pseudocapacitive mechanism or a battery‐type mechanism. In general, the specific capacitance of a pseudocapacitive electrode material exceeds that of a porous carbon‐based electrode making use of electrical double‐layer charge storage.^[^ ^59^ ^]^ As reported by Conway et al., the pseudocapacitance could be 10–100 times larger than that the capacitance of an EDLC.^[^ ^57^ ^]^

#### 2.1.3 HCs

HCs, which are also called battery‐EC hybrid device, provide an opportunity to take advantage of making use of the merits of both ECs (high power density) and batteries (high energy density) by combining a porously capacitive electrode (can either be EDLC or PC behavior) with a battery‐type electrode in the same cell.^[^ ^19^ ^]^ During charging (or discharging) process, electrolyte cations and anions move to (or separated from) the two electrodes, respectively, and ion accumulations (or separations) or rapid charge transfers occur at the capacitive electrode, while bulk redox/Faradaic reactions take place at the battery‐type electrode; in the meantime, the electrons flow pass through the external circuit (as illustrated in Figure 2d). There are four types of HCs (Figure 3c): i) metal‐ion HCs based on metal‐insertion electrode and an electrical double‐layer capacitance electrode (e.g., AC and graphene); ii) HCs based on metal‐insertion electrode and a pseudocapacitive electrode (e.g., MXene, MoS_2_); iii) acidic/alkaline HCs based on PbO_2_/Ni(OH)_2_ positive electrode and electrical double‐layer capacitance electrode; iv) HCs with redox electrolytes. Although being asymmetric, HC is different from the well‐developed “capacitive asymmetric EC,” in which both electrodes are capacitive (electric double‐layer capacitance or pseudocapacitance) but with asymmetric capacitive charge storage mechanism. As has been clarified by Liu and Jiang, four possible types of device configurations can be considered as HC (Figure 3d).^[^ ^20^ ^]^ Two general approaches have been taken to improve the energy density of HC,^[^ ^19^, ^20^ ^]^ namely, i) improving capacity and ii) extending voltage.

### 2.2 EC Electrolyte

**Figure 4:**
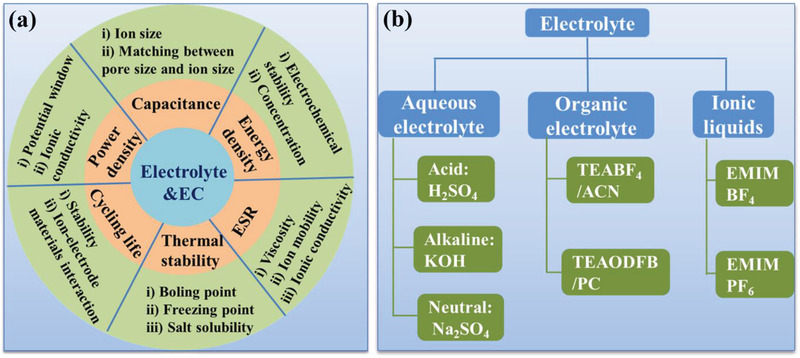
a) Effects of electrolyte on the EC performance. b) Classification of electrolytes for ECs.

Electrolytes, as one of the key components of ECs, provide the essential ionic conductivity and thus facilitate charge compensation on each electrode in the cell, thus determining the electrochemical performance of EC, such as the energy density, power density, capacitance, internal resistance, and cycle life (**Figure** **4**a). The energy density (*E*) and power density (*P*) of an EC are proportional to the *C* and the square of the operating voltage (*V*) (*E* = 1/2 *CV* ^2^ and *P* = *V* ^2^/4*R*_ESR,_ where *R*_ESR_ is the equivalent series resistance).^[^ ^1^ ^]^ Thus, having an electrolyte with the desired electrochemical stable potential window is an effective approach to simultaneously increase the *E* and *P*. Moreover, the interaction between the electrode materials and the electrolyte plays a critical role in the EC capacitance.^[^ ^32^ ^]^ For example, the matching between the size of electrolyte ions and the pore structure in carbon‐based electrode has a deep influence on the achievable capacitance (the details will be discussed in Section 3.1.3).

In general, the requirements for an ideal electrolyte to realize high‐performance EC are as follows: i) well‐matched with the electrode materials; ii) a wide working voltage window; iii) a high physicochemical and electrochemical stability; iv) a high ionic conductivity; v) a wide operating temperature range; vi) a low viscosity; vii) a low volatility and flammability; viii) low toxicity and environmentally friendly; and ix) low cost. To this end, a large variety of electrolytes in three types (Figure 4b and **Table** **1**): i) aqueous electrolyte, i.e., ions in water solvent, ii) organic electrolyte, salts in organic solvents, and iii) ILs, pure liquid salts, have been widely explored and significant achievements have been made with each of them, during the past several decades.^[^ ^60^, ^61^, ^62^, ^63^, ^64^ ^]^

**Table 1:** Summary of solvated ion size, ion conductivity, and thermal dynamic potential window of these commonly used aqueous, organic electrolyte and IL electrolyte for ECs.^[^ ^32^ ^]^

Aqueous electrolyte: acid (e.g., H_2_SO_4_ and H_3_PO_4_), alkaline (e.g., NaOH and KOH), neutral (e.g., Na_2_SO_4_ and Li_2_SO_4_) using water as solvent			
Ion	Solvated ion size [Å]	Ion conductivities [S cm^2^ mol^−1^]	Thermal dynamic potential windows [V]
H^+^	2.80	350.1	1.23
Li^+^	3.82	38.69	
Na^+^	3.58	50.11	
K^+^	3.31	73.5	
SO_4_^2−^	3.79	160.0	
PO_4_^3−^	3.39	207	
OH^−^	3.00	198	

Organic electrolyte: 0.65 m TEABF_4_ using propylene carbonate (PC) as solvent at 25 °C			
Ion	Solvated ion size [Å]	Ion conductivities [S cm^−1^]	Thermal dynamic potential windows [V]
TEA^+^	13	TEABF_4_: 0.0599	3.6
BF_4_^−^	3.3		

IL electrolyte: EMIMBF_4_ and BMIMTFSI at 25 °C (no solvent)			
Ion	Solvated ion size [Å]	Ion conductivities [S cm^−1^]	Thermal dynamic potential windows [V]
EMIM^+^	7.6	EMIMBF_4_: 0.015	3.5–4.0
BF_4_^−^	3.3		
TFSI^−^	7.9	EMIMTFSI: 0.088	

## 3 Porous Carbon‐Based Electrodes for High‐Volumetric‐Performance EDLCs

**Figure 5:**
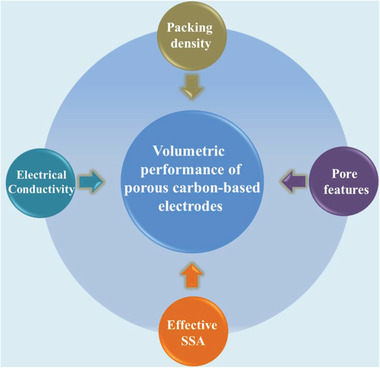
Factors influencing the volumetric performance of porous carbon‐base electrodes.

It is well known that the charge storage and volumetric capacitance of ECs are mainly dependent on the electrode materials employed. Various porous carbon‐based materials have been widely reported as electrodes for EDLCs, which store charge through highly reversible adsorption of electrolyte ions at the porous electrode/electrolyte interface and the electrolyte–ion transport in porous channels. As highly porous carbon materials usually exhibit nanopores and pore channels, the charge storage mechanism could become more complicated and obviously differ from what the traditional EDL model based on a planar surface predicts. Thus, from a materials science point of view, the volumetric performance of porous carbon‐based electrodes can be influenced by several factors, such as the packing density, effective SSA, pore features, and electrical conductivity. In this section, we will first discuss the various factors influencing the volumetric performance of porous carbon‐based electrode (**Figure** **5**) briefly, which will provide important guidelines for designing high‐volumetric‐performance EDLCs without sacrificing the gravimetric performance. An overview of recent advances on the development of porous carbon‐based electrodes for high‐volumetric‐performance EDLCs will be summarized.

### 3.1 Factors Impacting the Volumetric Performance of Porous Carbon‐Based Electrodes

#### 3.1.1 Packing Density

**Figure 6:**
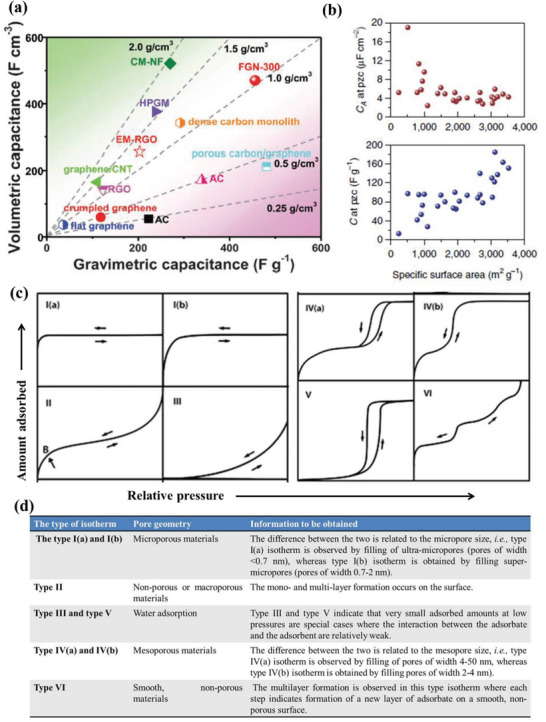
a) The *C*_vol_ and *C*_wt_ of previously reported carbon materials in aqueous electrolytes. Reproduced with permission.^[^ ^10^ ^]^ Copyright 2016, Royal Society of Chemistry. b) The area‐normalized capacitance (*C*_areal_, top) and the *C*_wt_ (bottom) of various types of porous carbon electrodes at the point of zero charge (PZC) versus the SSA. Reproduced with permission.^[^ ^66a^ ^]^ Copyright 2014, Nature Publishing Group. c) IUPAC classification of physisorption isotherms. d) Summary of information obtained from the different isotherms. c,d) Reproduced with permission.^[^ ^67b^ ^]^ Copyright 2015, The International Union of Pure and Applied Chemistry.

In general, the efficiency of an electrode for EDLC energy storage can be determined by its volumetric energy density against the whole EDLC stack (*E*_vol‐stack_) as follows^[^ ^8^ ^]^

(1) $$E_{\mathrm{vol}-\mathrm{electrode}}=C_{\mathrm{vol}}\times \Delta U^{2}/8$$

(2) $$C_{\mathrm{vol}}=C_{\mathrm{wt}}\times \rho$$

(3) $$E_{\mathrm{vol}-\mathrm{stack}}=E_{\mathrm{vol}-\mathrm{electrode}}\times f_{\mathrm{electrode}}$$

where *E*_vol‐electrode_ is the volumetric energy density of an EDLC electrode in relation to its volumetric capacitance (*C*_vol_, which is further associated with gravimetric capacitance [*C*_wt_] and the packing density of active materials [*ρ*]) and Δ*U*, and *f*_electrode_ is volume fraction of the electrode material. Obviously, broadening Δ*U* can be an effective approach to increase *E*_vol‐electrode_. To this end, as has been discussed in Section 2.2, various organic electrolytes and ILs that allow a large voltage window (up to 2.5–4.0 V) have been investigated for high‐energy‐density EDLCs.^[^ ^64^ ^]^ On the electrode side, apart from the *C*_wt_, the *ρ* also plays a critical role in determining the final *C*_vol_ (**Figure** **6**a).^[^ ^10^ ^]^ Therefore, achieving a high *C*_vol_ electrode requires both *C*_wt_ and *ρ* to be simultaneously maximized, where the inherent packing density/porosity and processing method of the carbon materials are necessary.

Generally, there is typically a contradictory relationship between *C*_wt_ and *ρ* for a selected carbon‐based electrode.^[^ ^34^ ^]^ On the one hand, a highly porous electrode with a large SSA will facilitate the rapid diffusion and transport of electrolyte ions for high *C*_wt_ (the light purple region in Figure 8a),^[^ ^10^ ^]^ but usually shows a low *ρ*, which results in plenty of empty spaces that could be flooded by the electrolytes, thereby raising the total weight of the device without contributing to the capacitance and lowering the total energy density normalized by the total weight of the entire device. On the other hand, a highly compact carbon‐based electrode with high *ρ* will reduce the ion‐accessible surface area and decrease ion transport rate, also leading to a relatively lower *C*_wt_ and poor rate capability (the light green region in Figure 6a).^[^ ^10^ ^]^ Thus, improving *C*_vol_ would need a class of carbon‐based electrode materials with optimum structures that can effectively increase the *ρ* while maintaining a high ion‐accessible surface area (or porosity). To this end, several pore‐controlling strategies,^[^ ^8^, ^35^, ^36^, ^65^ ^]^ including those for pore sizes,^[^ ^35^, ^65^ ^]^ pore connectivity,^[^ ^65d,e^ ^]^ and sub‐nanopores,^[^ ^36^ ^]^ have been employed to process carbon‐based electrode with high *ρ* while maintaining a porous architecture.

*Controlling of pore sizes*: Mechanical compression^[^ ^36^, ^65^ ^]^ and capillary compression^[^ ^65b,c^ ^]^ are two straightforward methods, which were employed to reduce the volume of macropores and mesopores inside a loosely packed electrode, with an increase in the corresponding *ρ*. For example, Ruoff et al. used mechanical compression to increase the *ρ* of an activated microwave‐expanded graphite oxide (aMEGO) electrode from 0.34 to 0.75 g cm^−3^, leading to a significant improvement of *C*_vol_ from 54 to 110 F cm^−3^ in an organic electrolyte.^[^ ^65a^ ^]^ In another example, by collapsing the macropores within hierarchical carbon nanocage (CNC) electrode by capillarity, Hu et al. increased the *ρ* up to 1.32 g cm^−3^, resulting in unexpectedly high *C*_vol_ of 233 F cm^−3^.^[^ ^65b^ ^]^ The two compression methods could not only be widely employed to densify the carbon‐based electrodes, ranging from ACs and CNTs to recently emerged novel graphene, but had shown the potential to use in large‐scale commercial applications. In addition, incorporating capacitive components into the macropores and mesopores, optimizing the micropore, and filling micropores with nanospacers are also effective strategies to increase *ρ* and the correspondingly volumetric performance as a result of the control in pore sizes.

*Controlling of pore connectivity*: For dense electrodes with a high *ρ*, the connected porous architecture featuring with superior pore connectivity will facilitate the fast ionic transport within the pores to offer access for the electrolyte ions to reach the surface of active materials, giving rise to high *C*_wt_ and *C*_vol_ simultaneously.^[^ ^65d^ ^]^ Thus, the importance of pore connectivity on volumetric capacitance has received considerable attention. For example, among the methods of engineering the pore connectivity and thus increasing the *ρ* of porous materials include: i) forming 3D pores with crossed nanoholes;^[^ ^65d^ ^]^ ii) tailoring aligned pores.^[^ ^65e^ ^]^ For instance, with an abundance of nanoholes distributed in the nanosheets to form a connective and porous structure, the holey graphene electrode with a high *ρ* of 1.2 g cm^−3^ delivered an improved *C*_vol_ compared with the common graphene film (53 F cm^−3^ vs 8 F cm^−3^).^[^ ^65d^ ^]^ To tailor the aligned pores, a vertically aligned reduced graphene oxide (VArGO) electrode with a high *ρ* of 1.18 g cm^−3^ was reported by Lee et al., made a simple rolling and cutting process.^[^ ^65e^ ^]^ The as‐fabricated VArGO electrode delivered a high *C*_vol_ of 171 F cm^−3^ in an aqueous electrolyte, because its porous and inter‐connected structure reduced the paths of the electrolyte ions in the graphene layers and promoted the rapid ion transport.

*Controlling of sub‐nanopores*: Generally, those sub‐nanopores within “porous and yet dense” electrode will only be passed by a reasonable “solution resistance,” arising from the restricted electrolyte diffusion together with issues of “wettability” and “collapsation,” leading to a much improved negative effect on the *C*_vol_, especially with the increase in the electrode in thickness. To better coordinate the sub‐nanopores and electrolyte ions, Li et al. developed a novel and effective method to increase the *ρ* of graphene‐based electrode by liquid‐mediated controlling of sub‐nanopores.^[^ ^36^ ^]^ Taking advantage of the unique colloidal assembly behavior of the chemically converted graphene (CCG), they obtained a “porous and yet dense” graphene electrode by capillary compression of the adaptive graphene hydrogel films in the presence of nonvolatile and volatile liquids. The *ρ* of these electrolyte‐mediated CCG (EM‐CCG) films was finely controlled from 0.13 to 1.33 g cm^−3^ by varying the ratio of the volatile and nonvolatile liquids, resulting in an unexpectedly high *C*_vol_ of 261 F cm^−3^.

The above discussions suggest that some of these pore‐controlling strategies can well balance the competitive relationship between the porosity and *ρ* of the “porous and yet dense” electrode, further realizing the high‐volumetric‐performance ECs. In light of this, the key design fundamentals to obtain high‐packing‐density electrode are pertaining to an adequate pore size, together with controls in pore size distribution (such as a reduction in the volumes of macropores and mesopores, a decrease in excess mesopores and macropores, and optimization in micropores), ensuring a pore connectivity and wettability, and harmonizing the sub‐nanopores and electrolyte ions. In this connection, 2D layered materials, such as graphene, MoS_2,_ and MXenes, could be among the most promising to obtain a high‐packing‐density electrode for high‐volumetric‐performance ECs, as their unique 2D structure is a great advantage for assembling a dense electrode while maintaining a porous structure together with the controllable/tunable pore features. For example, 1T MoS_2_^[^ ^65f^ ^]^ and MXene Ti_3_C_2_^[^ ^65g^ ^]^ film electrodes with densities of 5 and 3.6−3.8 g cm^−3^ have demonstrated considerably high *C*_vol_ of 700−900 and 900 F cm^−3^ in H_2_SO_4_ electrolyte, respectively.

#### 3.1.2 Effective SSA

As mentioned in Section 2.1.1, the electric double layer capacitance is generated from the reversible ions absorbed at porous electrode/electrolyte interface. As a consequence, the SSA and pore size distribution of active materials shall match the ion sizes, which are important for the produced *C*_wt_. To this end, tremendous efforts have been devoted to improving the *C*_wt_ by increasing the SSA of carbon materials, such as by use of porous carbon materials with high SSA ranging from 1000 to 3000 m^2^ g^−1^. However, several groups also found that the *C*_wt_ of modified carbons was limited even for the most porous samples possessing a very high SSA. For example, Ruoff et al. have reported that the *C*_wt_ increases together with the SSA of the porous carbon electrodes, but the area‐normalized capacitance gradually decreases to 4–5 µF cm^−2^, when the SSA is larger than 1500 m^2^ g^−1^ (Figure 8b).^[^ ^66a^ ^]^ The low area‐normalized capacitance observed in the highest SSA carbon materials are the main influence limiting the total available *C*_wt_ in porous carbon‐based electrodes. Barbieri et al. have attributed this capacitance saturation/plateau at ultrahigh SSA to a space charge constriction for charge accommodation inside the pore walls. Thus, as has been proposed by Chen et al., it is the effective SSA (ESSA) that is accessible to the electrolyte ions and eventually decides the *C*_wt_, and the ESSA is usually determined by both the total SSA and the pore size distribution of carbon materials based on the electrolyte ion sizes.^[^ ^66b^ ^]^ In addition, a high SSA may increase the risk of leading to decomposition of the electrolyte at the dangling bond positions, seriously deteriorating the energy and power densities of carbon‐based electrodes. In short, no linear relationship between SSA and *C*_wt_ could be established for the porous carbon‐based electrodes.

#### 3.1.3 Pore Features

Beside the above‐mentioned packing density and ESSA, there are other parameters that affect the volumetric performance of porous carbon‐based EDLC electrodes, such as pore features and electrical conductivity. It should be noted that the pores within a carbon material are not only closely associated with the packing density of the electrode, but their features including pore size, pore size distribution, pore connectivity, pore wettability, and pore tortuosity also impact on the ion transport and electron transfer, which eventually determine the volumetric capacitance. Thus, a comprehensive characterization of the pore feature/geometry is crucial in understanding how they affect the electrochemical performance of the “porous and yet dense” carbon‐based electrodes. As one of the most commonly used techniques, the gas sorption has been widely employed to measure the pore configurations of porous carbon,^[^ ^15^ ^]^ as well as other pore characteristics including SSA, average pore size, pore size distribution, and porous volume. They all can be estimated from the experimental gas sorption isotherms by using various theoretical models.^[^ ^67a^ ^]^ The International Union of Pure and Applied Chemistry (IUPAC) has published an updated classification of the types of physisorption isotherms in 2015, shown in Figure 6c, and the corresponding information obtained from different isotherms are summarized in Figure 6d.^[^ ^67b^ ^]^

Note that it is necessary to carefully select a probe gas and an appropriate model for evaluating the pore geometry and SSA of porous carbons, especially when micropores (<2 nm) are involved.^[^ ^67c^ ^]^ To this end, N_2_, Ar, and CO_2_ gas sorptions are among the suitable probes in assessing the mesopores/macropores (>2 nm), super‐micropores (0.7–2 nm), and ultra‐micropores (<0.7 nm), respectively. Regarding the selection of appropriate models, apart from the classical Brunauer–Emmett–Teller (BET) equation for macro/mesoscopic materials, various density functional theory (DFT)‐based models and simulation techniques from the microscopic level can also be applied to analyze the data of gas sorption experiments with improved accuracies.^[^ ^68a–c^ ^]^ Moreover, other techniques (such as scattering methods) have been used to obtain level of porosity and understand pore configurations of different porous carbons,^[^ ^69^ ^]^ showing a good agreement with gas adsorption. This has contributed to further understanding on the correlation between the electrochemical performance and pore nature.

In addition, considering that porous electrode materials would experience likely structural changes (such as pore geometry and structure) during electrochemical process, researchers have recently developed in situ techniques, including in situ small‐angle X‐ray scattering (SAXS), in situ NMR spectroscopy, and in situ electrochemical quartz crystal microbalance (EQCM), which have been used in the real‐time detection of structural variations as a function of voltage in the electrochemical process. For more details on the using of these in situ characterization methods, readers are referred to recent reviews by Lee and Chen^[^ ^18d^ ^]^ and Simon et al.^[^ ^15^ ^]^

In the following sections, various pore features that influence the volumetric performance of porous carbon‐based electrodes will be discussed.

a) Pore size

It is well known that the pore size of a porous carbon‐based electrode has a critical impact on its ultimate volumetric performance, because it largely determines the ion adsorption behavior. In general, the SSA mainly arises from a complex network of internal pores with different sizes, including macropores (>50 nm), mesopores (2.0–50 nm), and micropores (<2.0 nm), and the three different pore sizes have different functions in the charging/discharging process of EDLC.^[^ ^10^ ^]^ Macropores can serve as ion‐buffering reservoirs, which provide avenues for ions transport from the external electrolyte into the interior surface of active materials. The medium‐sized mesopores could act as ion highways and facilitate the adsorbate accessibility by offering effective transport channels for the diffusion of electrolyte ions into the bulk of the electrode. Compared to the macropores and mesopores, micropores have been proven to be more effective in charging accommodation of the adsorption‐based mechanism through controlled diffusion and molecular sieve effects. Thus, an adequate pore size together with appropriate pore size distribution is believed to play a more important role in achieving a high‐volumetric‐performance electrode than by a simply high SSA. Taking ACs as an example, they can be made to possess very large SSAs up to 3000 m^2^ g^−1^, which have been the most used carbon‐based electrodes for commercial EDLCs, but most of them only exhibit *C*_wt_ of 130–230 F g^−1^ and *C*_vol_ of 60–160 F cm^−3^ in organic electrolytes.^[^ ^70^ ^]^ The insufficient capacitance performance is largely attributed to their moderately broad pore size distribution from micropore to macropore, in which the micropores (<0.5 nm) are considered to be inaccessible to electrolyte ions, resulting in a low degree of porosity utilization. There is a long‐held theory stating that porous carbon with a pore size smaller than the size of solvated electrolyte ion does not take part in the formation of electric double‐layer capacitance, and thus is considered useless.^[^ ^71^ ^]^ In other words, pores can contribute to the capacitance only when their diameters match the size of electrolyte ions. Indeed, Largeot et al. have experimentally investigated the relationship between pore size in carbon materials and ion size for an EDLC, and revealed that the optimum pore size resulting in the maximum electrical double‐layer capacitance was very close to the size of electrolyte ion.^[^ ^72a^ ^]^ Due to the ion sieving effect, pore sizes either larger or smaller than those of electrolyte ions can lead to a significant drop in capacitance.

**Figure 7:**
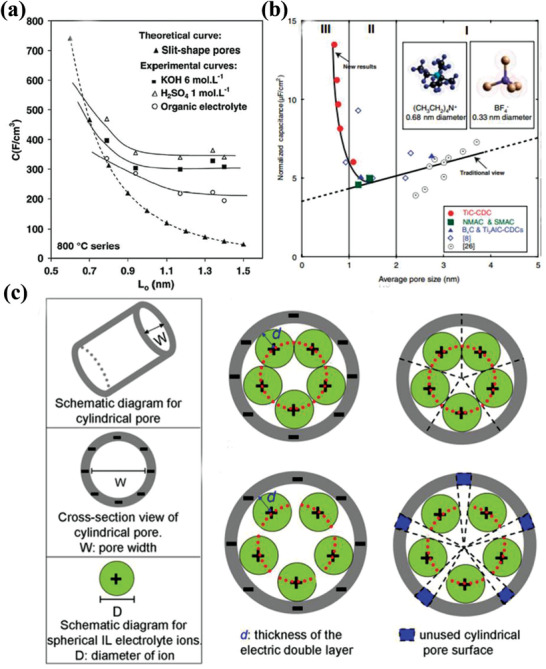
a) Relationship between the *C*_vol_ (theoretical and experimental) and the pore size in aqueous electrolyte or organic electrolyte for ACs. Reproduced with permission.^[^ ^72b^ ^]^ Copyright 2006, Elsevier. b) Plot of *C*_areal_ normalized by SSA for the carbons in this study and in two other studies with identical electrolytes. Reproduced with permission.^[^ ^72c^ ^]^ Copyright 2010, The Royal Society Publishing. c) Schematic diagrams of a model based on the spherical IL electrolyte ions and carbon materials with cylindrical pore surface. Reproduced with permission.^[^ ^66b^ ^]^ Copyright 2013, American Chemical Society.

Generally speaking, the sizes of bare ions and correspondingly solvated ions vary from a few to tens of Å for these commonly used electrolytes. For example, the bare tetraethylammonium cation shows a small size of 0.68 nm, but its solvation shell in acetonitrile will increase the solvated ion close to 1.3 nm. To this end, porous carbon materials with large micropores and mesopores are of importance in maximizing the capacitance. As shown in **Figure** **7**a, the efficiency of pore filling in double layer formation is optimal when the pore size is close to 0.7 nm in aqueous media and 0.8 nm in organic electrolyte,^[^ ^72b^ ^]^ again suggesting that for such sizes, micropores are electrochemically accessible by electrolyte ions to form an electrical double‐layer, and they appear to be the most suitable candidates for maximizing the capacitance. More recently, several prominent studies, both experimental and theoretical, have demonstrated that high capacitance can also be obtained by using microporous carbons with sub‐nanometer pores in various electrolyte systems.^[^ ^72^, ^73^ ^]^ For example, Simon and Gogotsi observed anomalous increase both in *C*_wt_ and *C*_vol_ on a carbide‐derived carbon (CDC) electrode with the pore size below 1 nm by making full use of the tunable pore structure and pore size distribution of CDCs (Figure 7b).^[^ ^72c^ ^]^ In addition, the sub‐nanometer‐sized pores ranging from 0.55 to 0.70 nm by liquid‐mediated engineering ensure the electrolyte ions to insert into the smaller pores, giving rise to ultrahigh *C*_wt_ and *C*_vol_ in both aqueous and organic electrolytes. However, it should be noted that although sub‐nanometer pores can contribute to high *C*_wt_ and *C*_vol_, they could also lead to poor rate capability and insufficient power performance.^[^ ^36^ ^]^

b) Pore size distribution

Pore size distribution also has a crucial impact on the electrochemical performance of carbon materials. First, as discussed in Section 3.1.2, the pore size distribution of carbon materials is one of the key factors in determining the ESSA, which is accessible to the electrolyte ions and eventually decides the *C*_wt_ of porous carbon‐based electrodes. Second, it has been found that a narrow pore size distribution is favored, because it can reduce ion scattering and consequently improve the electrode kinetics, resulting in an increase in the energy‐stored capability and power performance.^[^ ^74^ ^]^ Indeed, optimization of the pore size distribution in carbon materials, studies conducted using both theory and experiment, has been demonstrated to be an effective strategy to increase their capacitive performance. Previous works have also reported that the pore size distribution of porous carbon materials can be controlled by using different carbon sources and adjusting the activation parameters.^[^ ^64^, ^66^, ^75^ ^]^ For example, according to Chen et al., various sp^2^ carbon materials with controlled pore size distribution in association with different SSA were synthesized at large scale by employing different carbon sources and preparation methods.^[^ ^66b^ ^]^ They further proposed a theoretical model using the slit/cylindrical NL‐DFT approach (Figure 7c), and the results were well consistent with the experimental results for the influence of pore size distribution (PSD) and ESSA on the capacitance performance. The proposed model can also be extended to porous carbon materials with bimodal porous distribution, which show comparatively narrow pore size distributions of micropores and mesopores. Finally, the pore size distribution is related to the pore regularity of porous carbon materials, which is also one critical influencing factor that needs to be considered for their ultimate electrochemical performance. In contrast, the presence of an increasing level of defects will decrease the regularity of the pores. Based on the above‐mentioned discussions, considerable research efforts need to be devoted to understanding and optimizing the pore size distribution to improve pore utilization, moving from disordered hierarchical pores to ordered micropores and mesopores; thus, the porous carbon‐based electrode with an improved overall performance can be obtained.

c) Pore connectivity

Besides the pore size and pore size distribution, pore connectivity has also shown an important influence on the volumetric performance, because it plays a key role to balance the porosity level and the *ρ* of the “porous and yet dense” carbon‐based electrode. For dense electrode with a high *ρ*, an interconnected porous architecture possesses the highly continuous network of open channels, ensuring a rapid and efficient transport for electrolyte ions through the entire network and leading to an excellent *C*_wt_ (which is further associated with a high *C*_vol_).^[^ ^3^, ^9^, ^35^ ^]^ In addition, the pore connectivity in association with a continuous ion transport network can guarantee a low ion interfacial resistance, which further improves fast charging/discharging capability and high‐rate performance.

**Figure 8:**
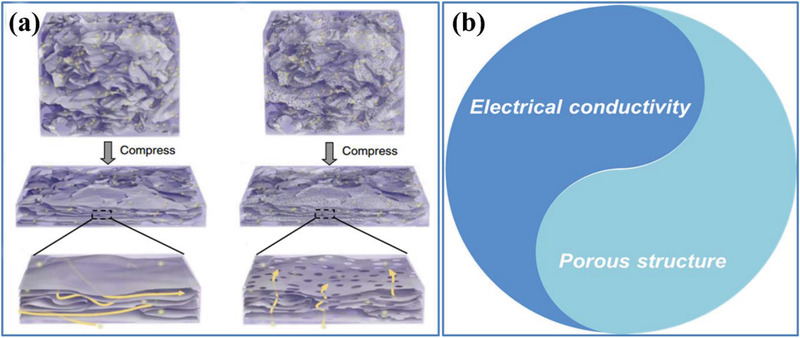
a) Schematic illustration of the HGFs as a material for EC electrodes. Reproduced with permission.^[^ ^35^ ^]^ Copyright 2014, Nature Publishing Group. b) The trade‐off relationship between electrical conductivity and pore structure.

As summarized in Section 3.1.1, forming 3D pores with crossed nanoholes^[^ ^65d^ ^]^ and tailoring aligned pores^[^ ^65e^ ^]^ are two commonly used methods for engineering of pore connectivity to improve the *C*_vol_ of a “porous and yet dense” electrode. For example, Duan et al. used the former strategy to report a 3D holey graphene framework (HGF) with a hierarchical porous structure as a high‐performance binder‐free EDLC electrode, which delivered a high *C*_wt_ of 298 F g^−1^ and *C*_vol_ of 212 F cm^−3^ in an organic electrolyte. The impressive high performance is attributed to the unique architecture of the HGFs showing several important demands for an ideal EDLC electrode as follows (**Figure** **8**a).^[^ ^35^ ^]^ i) The 3D porous monolithic HGF resulted from these highly interconnected and interlocked graphene sheets with numerous nanopores has a high SSA of up to 1560 m^−2^ g^−1^. ii) Compared with these ultra‐small micropores in activated graphenes that are considered to be inaccessible to electrolyte ions, nanopores in the HGFs are sufficiently large and well integrated into an open and continuous porous architecture, which can facilitate the rapid ionic transport within the pores to offer access for ions to reach the surface of active materials. iii) The HGFs are fully hydrated and thus permit direct exchange of various electrolytes to guarantee the entire pores fully wetted by the electrolyte ions. iv) The HGF films also show an outstanding conductivity of 1000 S m^−1^. In addition, Hu et al. employed a scalable and single‐step method to obtain holey graphene nanosheets, where the 2D nanoholes (>1.3 nm) can be viable pathways for the access and penetration of electrolyte ions (EMI:TFSI)^[^ ^65d^ ^]^ Furthermore, due to an abundance of nanoholes (0.9–1.7 nm) distributed in the nanosheets showing great potential to form connective and porous structures, the holey graphene electrode with a high *ρ* of 1.2 g cm^−3^ delivered an improved *C*_vol_ compared with the common graphene film (53 F cm^−3^ vs 8 F cm^−3^).

Regarding tailoring aligned pores, a previous report has demonstrated that vertically oriented graphene with a suitable porous and connectivity structure can build up a high‐speed and short‐cut bridge between active materials and current collector to facilitate the rapid ion mobility and electron transport.^[^ ^74b^ ^]^ Inspired by this, a vertically aligned reduced graphene oxide (VArGO) electrode with a high *ρ* of 1.18 g cm^−3^ was prepared using a simple rolling and cutting process.^[^ ^65e^ ^]^ The as‐fabricated VArGO electrode delivered a high *C*_vol_ of 171 F cm^−3^ in an aqueous electrolyte. Its volumetric performance was well maintained even as the film thickness of VArGO electrode increased.

It should be noted that rather limited information can be obtained on the connectivity of freely accessible mesopores by physisorption analysis alone. Thus, coupling the state‐of‐the‐art gas adsorption analyses with complementary characterization techniques (e.g., advanced imaging techniques based on electron tomography and 3D rotation electron diffraction or simulation tools) would be required, in order to get a full picture of the pore connectivity. For example, Thommes et al. used these techniques to reveal the connectivity of mesopores.^[^ ^74c,d^ ^]^ 3D reconstructions were obtained, demonstrating an interconnected micro‐ and mesopore system with hierarchical structure. Mercury intrusion porosimetry, NMR, transmission electronic microscopy (TEM), and in situ X‐ray and neutron scattering (SAXS) have also been demonstrated useful in providing important complementary information on pore connectivity of porous carbons.^[^ ^74e,f^ ^]^

d) Pore wettability

The surface wettability of an electrode should also be considered when designing porous carbon materials for ECs.^[^ ^64^, ^75^ ^]^ In other words, those pores within porous carbon have to be engineered to possess suitable wetting capabilities toward a given electrolyte (or called electrolyte‐philic). A superior surface wettability will not only affect the contact surface between carbon materials and electrolyte and facilitate the adsorption and transportation of the electrolyte ions, leading to improved energy storage capabilities,^[^ ^8^, ^11^ ^]^ but also decreases the ion transport resistance to enhance the rate performance. For example, Kim et al. studied the influence of surface wettability on the capacitance of CNT electrode by introducing various functional groups on their surface.^[^ ^75b^ ^]^ They showed that the introduction of surface carboxyl groups resulted in a 3.2 times higher capacitance because of the improved electrolyte‐philic of the electrode surface in an aqueous electrolyte. The surface chemistry (e.g., surface functional groups and surface charge) can enable different wettability for aqueous electrolytes, organic electrolytes, and ion liquid (IL) electrolyte. Taking solvated HGF as an example,^[^ ^35^ ^]^ because of the highly hydrated micropores in holey graphene sheets, the HGF can be directly exchanged with various electrolytes (including aqueous electrolyte [1 m KOH], organic electrolyte [1 m EMIMBF_4_/AN], and neat IL electrolyte [EMIMBF_4_]); consequently, the entire surface area shows a high wettability and is accessible by electrolyte ions and thus electrochemically active, contributing to an improved volumetric capacitance and rate capability. However, on the one hand, a comparison of HGF‐ECs with organic electrolyte (1 m EMIMBF_4_/AN) and neat IL electrolyte (EMIMBF_4_) indicates that the device delivers a slightly higher volumetric capacitance and rate capability in organic electrolyte than neat IL electrolyte. Such difference is attributed to a higher wettability toward the organic electrolyte in liquid state. A higher mobility can be obtained in the presence of micropores, when compared with the IL, because of the conflicting forces at the sub‐nanoscale.^[^ ^36^ ^]^ On the other hand, the volumetric capacitance achieved in organic electrolyte (1 m EMIMBF_4_/AN) is about 4% lower than that in aqueous electrolyte (1 m KOH), which could be resulted from the different surface wettability in association with the distinct ability of electrolyte ion adsorption and transportation in two kinds of electrolytes.

Similar results have been demonstrated by Li et al.^[^ ^36^ ^]^ and Zhang et al.,^[^ ^9^ ^]^ where liquid electrolyte‐mediated chemically converted graphene (EM‐CCG) film and boron‐, nitrogen‐, and phosphorus ternary‐doped holey graphene hydrogel (BNP‐HGH) film were directly used as the bind‐free electrodes for ECs in aqueous electrolyte and organic electrolyte, respectively. DFT calculations revealed that the surface chemistry (e.g., surface functional groups and surface charge) was largely related to these doped heteroatoms (N, P, and S), leading to different wettabilities between the aqueous electrolyte and organic electrolyte. The effect of wettability is recognized by the indirect measurement of electronic conductivity and the capacitance of porous carbon‐based electrodes. More recently, a new method of deciding the pore‐filling ratio in carbon‐based electrodes was established, by employing propylene carbonate as the electrolyte solvent, reported by Nishihara et al.^[^ ^76a^ ^]^

In addition, the surface chemistry has also shown significant influence on the stable working potential window of ECs. Taking porous carbon electrodes working in aqueous electrolyte as an example, widening in the stable potential window could be obtained by increasing the over‐potentials of hydrogen evolution reaction (HER) for cathode and oxygen evolution reaction (OER) for anode. A modulation in surface chemistry of porous carbon materials by introducing heteroatom‐containing functional groups can regulate the electronic properties, giving rise to an improvement of electrochemical performance. It inevitably promotes the HER activity (make the intermediate state reversible),^[^ ^76b^ ^]^ which is not beneficial to the widening potential window. To this end, as has been reported by Pan et al., heteroatom‐rich micropore carbon cloth (CC) electrode can be modified by introducing Na^+^ into the electrode surface, in which the Na‐containing functional groups CC (Na‐CC) exhibits an expanded negative potential window, as they inhibit the adsorption of H^+^.^[^ ^76c^ ^]^ As a result, a high‐volumetric‐performance asymmetric supercapacitor is developed by using Na‐CC as negative electrode and CC as the positive electrode within aqueous electrolyte, exhibiting an extended and stability working potential window of 2.1 V. In addition, to investigate the electrochemical stability of the EM‐CCG electrodes under a working voltage of 3.5 V, Li et al. fabricated an EC with an IL electrolyte (EMIMBF_4_) and tested the stability using the voltage holding method.^[^ ^36^ ^]^ Due to the superior surface wettability (electrolyte‐philic) of the EM‐CCG films derived from the selective removing/remaining of the volatile/nonvolatile solvent under capillary compression, over 95% capacitance was retained in the voltage holding at 3.5 V at room temperature for 300 h.

e) Pore tortuosity

Apart from the above‐mentioned pore features, concerning the pore tortuosity, anisotropic pores preferentially aligned in the ion transport direction have shown an influence on the electrochemical performance of “porous and yet dense” carbon electrode. It is closely related to the tortuosity of the ion and electron transport paths, which largely determines the charging/discharging ability and rate performance of EDLCs.^[^ ^77^ ^]^ In the present context, the tortuosity (*τ*) can be measured by a ratio that characterizes the convoluted pathways of electrolyte ion diffusion of a porous electrode, defined as *τ* = *ε*(*D/D*_T_), where *ε* is the porosity (pore volume fraction), *D* represents the conductivity of the electrolyte, and *D*_T_ refers to the macroscopic diffusivity.^[^ ^77a,e,f^ ^]^ An ideal electrode structure shall show straight and aligned channels, which lead to a low tortuosity (lower to 1) to ensure high ion/electron diffusivity. However, commonly used electrodes of 30–40% porosity typically have *τ* = 3−5.^[^ ^78a^ ^]^ Moreover, the value can be further increased in the thick electrode with a high mass loading or dense electrode with a high packing density, due to the improved difficulty of ion and electron transport. Thus, it would be necessary to minimize/lower the tortuosity of porous carbon electrode, in which the transport of ions and electrons can be accelerated by reducing the diffusion distance, leading to an improved capacitance performance and rate capability.^[^ ^78b^ ^]^

Recently, two general strategies have been explored to lower tortuosity in porous carbon electrodes: i) constructing electrodes with aligned pores or active materials along the ion transport direction by external or internal magnetic fields;^[^ ^77e–g^ ^]^ ii) carbonizing multichanneled natural wood to obtain 3D wood‐carbon monolith along the thickness direction.^[^ ^77a–d,g,h^ ^]^ For example, Chiang et al. used magnetic control of sacrificial phases to introduce anisotropic and low‐tortuosity arrays of aligned pores preferentially oriented in the primary transport direction of porous electrodes.^[^ ^77f^ ^]^ Subsequently, they further reported an emulsion‐based and magnetic‐alignment method to produce low‐tortuosity and ultrahigh‐capacity electrodes.^[^ ^77i^ ^]^ On the other hand, by directly carbonizing the aligned, multichanneled natural wood, Hu et al. obtained a highly conductive, lightweight, and low‐tortuosity 3D wood‐carbon monolith,^[^ ^77^, ^78^ ^]^ which has been successfully used as the active material or current collected for energy storage devices (supercapacitors,^[^ ^77a^ ^]^ lithium‐ion battery,^[^ ^77b^ ^]^ and lithium–sulfur battery^[^ ^77c^ ^]^). More discussions on the advances of low‐tortuosity carbon electrodes with improved electrochemical performance will be summarized in “d) aligned pores” in Section 3.3.

In short, the pore features of carbon‐based electrodes, including pore size, pore size distribution, pore connectivity, pore wettability, and pore tortuosity, all play critical roles in the final electrochemical performance, because they are closely related to the processes of electron transfer and ion transport. Thus, significant research efforts have been devoted to optimizing the key pore features in order to increase the pore utilization, where, e.g., to move from disordered hierarchical pores to ordered mesopores and micropores (ordered hierarchical pores). For EDLC electrodes, among the ideal pore features shall include: a) controlled pore sizes matching those of electrolyte ions; b) narrow pore size distribution to reduce ion scattering and consequently improve the electrode kinetics; c) superior pore connectivity for continuous ion transport network to facilitate the rapid ion transport and thus improve ion transport behavior; and d) high level of pore wettability to decrease the ion transport resistance and increase the contact surface of the carbon materials and electrolyte. Indeed, it has been demonstrated that an appropriate hierarchical porous structure with high SSAs, well‐controlled pore sizes with arrow pore size distribution, superior pore connectivity, and wettability, and high electrical conductivity is effective in improving both the energy and power densities.

#### 3.1.4 Electrical Conductivity

The electrical conductivity of active materials in porous electrodes is important with respect to their electrochemical performance in terms of internal resistance, capacitance, charging/discharging capability, and power density. During the charging or discharging process, electrons accumulate on or depart from the surface of positive and negative electrodes. The electrolyte–electrode interface within the porous carbon‐based electrode is balanced by the counter‐ions from the electrolyte to store charges/energies. Thus, an improvement in electrical conductivity of the carbon‐based electrodes by adding conducting materials is beneficial for achieving an optimum *C*_wt_ and energy density of an EDLC. For example, Bandosz et al. introduced about 5 wt% commercial graphene into nanoporous sodium‐salt‐polymer‐derived carbon to improve its electrical conductivity; thus, the degree of the pore space utilization for energy storage increased proportionally, because small pores (< 0.7 nm) that were not previously being electrical were found as very active for double layer capacitance.^[^ ^79a^ ^]^ More recently, there are research interests that focus on preparing free‐standing/binder‐free porous carbon‐based electrodes with outstanding intrinsic electrical conductivity to obtain high *C*_wt_.

Although significant research progress has been made with the carbon‐based electrodes with higher *C*_wt_ by improving their electrical conductivity, it should be noted that there is usually a trade‐off relationship between electrical conductivity and pore structure for the selected porous carbon material (Figure 8b). On the one hand, carbon materials with excellent electrical conductivity usually need a high degree of graphitization by a high temperature treatment, which easily leads to a low SSA and an undeveloped pore structure owing to the pore collapse, suppressing the diffusion and transport of electrolyte ions. On the other hand, a highly porous structure could offer a high SSA and favorable channels for ion diffusion; however, this usually results in a poor electrical conductivity because of the low graphitization degree, which decreases the transfer rate of electrons. Therefore, it is advised to optimize the transfer rate of electrons and diffusion/transport of electrolyte ions through pore‐engineering design of the porous carbon materials to maintain the balance between pore structure and electrical conductivity to maximize the electrochemical performance. For instance, the construction of 3D nanostructured carbon composites/hybrids by introducing 1D carbon nanomaterials (e.g., CNTs and carbon nanofibers (CNFs)) that show a large aspect ratio and an excellent electrical conductivity into 2D materials (e.g., graphene and MXene) is a promising strategy,^[^ ^79b^ ^]^ which can achieve a balance between pore structure and electrical conductivity, giving rise to the improvement of both *C*_wt_ and *C*_vol_.

### 3.2 Interconnections among Factors in Volumetric Performance

**Figure 9:**
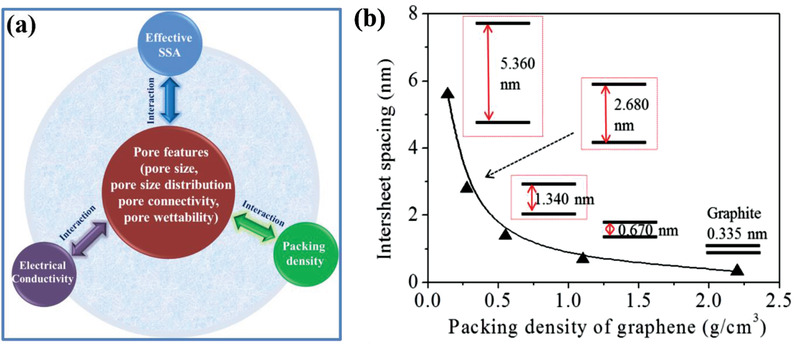
a) Schematic diagram of the interactions between pore features with ESSA, *ρ*, and electrical conductivity. b) Theoretical estimation of the relation between the intersheet spacing and the *ρ* of graphene in a face‐to‐face arranged assembly. Reproduced with permission.^[^ ^36^ ^]^ Copyright 2013, American Association for the Advancement of Science (AAAS).

It should be pointed out that the four factors discussed above on influencing the volumetric performance of porous carbon‐based electrodes may not be independent, but can have close interactions among them. Their interrelation/interaction is summarized in **Figure** **9**a, where the pore feature (including pore size, pore size distribution, pore connectivity, and pore wettability) is the core factor, where there is a competitive relationship with *ρ*, ESSA, and electrical conductivity. As suggested by Yang et al., there is typically a contradictory relationship between the *ρ* and the level of porosity. On the one hand, a highly porous electrode with a low *ρ* usually possesses an abundance of voids/spaces because of excessive mesopores and macropores. On the other hand, a highly compact structure would be needed to obtain an ultrahigh *ρ*, which can give rise to a small pore size or narrow channels.^[^ ^8^ ^]^ Taking graphite and AC as examples, due to the dense face‐to‐face parallel stacking from 2D graphene layers, graphite is a rather compact carbon material with a high *ρ* of ≈2.2 g cm^−3^, and there is a narrow average inter‐planar spacing of ≈0.335 nm. In contrast, the *ρ* of commercial AC‐based electrode decreases to about 0.5 g cm^−3^ as the average inter‐planar spacing (i.e., pore size between two adjacent graphene layers) of graphene film increases to 1.47 nm (Figure 9b). Concerning the interaction between ESSA and pore size, the former is largely determined by the pore size distribution in porous carbon materials. A narrow pore size distribution together with a smaller micropore size can be effective in increasing the ESSA, resulting in a high volumetric performance. For instance, Kyotani et al. reported that zeolite‐templated porous carbon with 3D connected 1.2 nm micropores exhibited a high ESSA and *C*_vol_ (83 F cm^−3^) as well as a high rate performance.^[^ ^79c^ ^]^ As has been discussed in Section 3.1.4, a similar trade‐off relationship between electrical conductivity and pore structure can also be found in the selected porous carbon materials.

In short, when addressing one aspect of the porous structures in carbon‐based electrodes, one shall well consider alleviating one or more of other competing issues at the same time. Therefore, it would be highly desirable to explore an integrated strategy to design “porous and yet dense” carbon‐based electrodes toward high‐volumetric‐performance ECs, in which the engineering of pores should be the first and core consideration. In the following section, from a pore‐engineering perspective, the recent advances for high‐volumetric‐performance EDLCs obtained by the rational design and development of porous carbon‐based electrodes will be visited.

### 3.3 Advances in Porous Carbon‐Based Electrodes for High‐Volumetric‐Performance EDLCs

Over the past three decades, considerable efforts have been made to improve the volumetric performance of EDLCs by exploring various porous carbon materials due to their high and tunable SSA, well‐controlled/tunable pore features (pore size, pore size distribution, pore connectivity, and pore wettability), excellent electrical conductivity, abundant surface functionalities, and good thermal, physical, and chemical stability. Moreover, the incorporation of pseudocapacitive materials (such as transition metal oxides and conductive polymers) into various dimensional porous carbon materials to obtain multicomponent composites has been shown to further increase the overall electrochemical performance by taking full advantage of the synergistic effects between the individual components, which will be discussed in Section 4.2 in detail. With the steady advance in nanotechnology, a large number of new porous carbon materials with well‐defined nanostructures and functionalized pores, such as ACs, CDCs, biomass‐derived carbons, CNTs, CNFs, graphene, and their composites, have been synthesized by various approaches.^[^ ^10^, ^11^, ^14^ ^]^ Although they have been shown to exhibit high *C*_wt_ and thus high gravimetric energy and power densities, the correspondingly volumetric performance is relatively insufficient owing to their low *ρ* (<0.8 g cm^−3^). Thus, it would be imperative to develop the advanced porous carbon‐based electrodes with both desirable *C*_wt_ and *C*_vol_ from a pore‐engineering viewpoint, as it plays a key role to the design of dense electrodes with different porous architectures (as we discussed in Sections 3.1.3 and 3.2).

For porous carbon‐based electrodes, surface charge generation involves dissociation and ion adsorption from both the electrolyte and crystal lattice defects, thus no charge transfers across the electrode/electrolyte interface and the energy storage is a true capacitance. To facilitate the surface charge generation so as to maximize the resulted capacitance performance, morphological and structural control of the porous carbon materials to create suitable pore architecture/feature is critical. Basically, the pore volume percentage in a carbon‐based electrode indicates the total electrolyte uptake, and the pore size and pore size distribution largely determine the diffusion resistance of electrolyte (i.e., the easiness of ions transport) and how much electrolyte can be efficiently utilized without wasting any. In addition, one has to take note that since the double‐layer capacitance occurs only on the surface/near‐surface of an active material, the thickness should theoretically be as small as possible in order to render the whole electrode “fully active.” This is also relevant to how porous the electrode is. On the other side, the structural integrity and stability is affected by the level of porosity.

Pore engineering is an effective approach in achieving high‐volumetric‐performance EDLCs, where one shall consider both individual parameters as well as the synergetic interplay of several key factors including the pore volume, pore size, pore size distribution, pore connectivity, pore wettability, and pore tortuosity. While the geometrical arrangement of pores in the electrode holds the most preliminary effect on the smoothness of electrolyte/ions transport and the contact effectiveness of the ions and the electrode surface, it is reasonable to categorize the porous carbon‐based electrodes based on pore structures and analyze their capacitance efficacy. Overall, the pores can be individually and separately distributed in the electrodes (denoted as separated pores), or interconnected with one another to form 3D continuous nanochannels (denoted as interconnected pores), or hybridized with both (denoted as hybrid pores), or anisotropic pores preferentially oriented in the primary transport direction (denoted as aligned pores). With AC being the most common example with random pores created inside through high‐temperature annealing or physical/chemical activation, the pore size and pore size distribution can be evaluated using the BET technique and correlated with the ions accessibility. Among the remaining challenges are how the pores (especially micropores) are arranged and whether they are fully distributed throughout the whole bulky carbon structure. There are also some uncertainties on whether the electrode has been fully utilized without wasting any of the active material. The pore engineering of carbon‐based electrodes requires precise controls of pore size, pore size distribution, pore connectivity, and pore wettability, which are the structural parameters dominating the volumetric performance. Several strategies have been developed over the past three decades, which will be summarized and discussed separately (**Table** **2**). There is an explosion of publications in this exciting field of exploring porous carbon‐based electrodes for high‐volumetric‐performance EDLCs. Therefore, these references provided here are not meant to be comprehensive, but they are representative.

a) Separated pores

**Table 2:** Summary of “porous and yet dense” electrodes based on carbon materials discussed in this review

Electrode Materials^a)^	Preparation methods for electrode	Specific surface area [SSA, m^2^ g^−1^]	Packing density [g cm^−3^]	Working voltage window [V] (electrolyte)	Maximal volumetric capacitance [*C*_vol_, F cm^−3^]	Maximal volumetric energy density [*E*_vol_, W h L^−1^]	Refs.
BNP‐HGH	Modified hydrothermal reduction with BPO_4_ and NH_4_BF_4_ solution	498	0.67	3.5 (Organic electrolyte: EMIMBF_4_/AN)	234	57.4 (device)	^[^ ^9^ ^]^
HGF	Hydrothermal reduction with diluted H_2_O_2_ solution	1560	0.71	3.5 (Organic electrolyte: EMIMBF_4_/AN)	212	49 (device)	^[^ ^35^ ^]^
EM‐CCG	Capillary compression of the CCG in the presence of nonvolatile and volatile liquids	166.8	1.25	3.5 (Organic electrolyte: EMIMBF_4_/AN)	261.3	59.9 (device)	^[^ ^36^ ^]^
aMEGO	Microwave irradiation followed by KOH activation, and mechanical compression	707	0.75	3.5 (Organic electrolyte: TEABF_4_/AN)	110	48 (device)	^[^ ^65a^ ^]^
CCNC	In situ MgO template method, and capillary compression	2561	1.32	4.0 (IL electrolyte: EMIMBF_4_	233	114 (electrode)	^[^ ^65b^ ^]^
h‐graphene	Controlled heating in air	658	1.2	4.0 (IL electrolyte: EMIMTFSI	53	12 (electrode)	^[^ ^65d^ ^]^
VArGO	Hand rolling and cutting	123.8	1.18	0.7 (Aqueous electrolyte: KOH	171	7.43 (electrode)	^[^ ^65e^ ^]^
MAC‐A	Template method followed by KOH activation	2222	0.93	2.4 (Organic electrolyte: NEt_4_BF_4_/AN)	145	29 (electrode)	^[^ ^79d^ ^]^
ALG‐C	Carbonization followed by acid etching	273	0.91	0.7 (Aqueous electrolyte: H_2_SO_4_	180	9.1 (electrode)	^[^ ^85^ ^]^
CMK‐3	CO_2_ activation	984	NA	1.0 (Aqueous electrolyte: KOH	43	NA	^[^ ^80^ ^]^
a‐MEGO	Microwave irradiation followed by KOH activation	2400	NA	3.5 (Organic electrolyte: TEABF_4_/AN)	60	23 (device)	^[^ ^82^ ^]^
ac‐Gr/SWCNT	Electrostatic self‐assembly of GO and SWCNTs, followed by KOH activation	652	1.06	4.0 (IL electrolyte: EMIMBF_4_	211	117.2 (electrode)	^[^ ^90^ ^]^
HPGM	Hydrothermal followed by evaporation‐induced drying	367	1.58	2.5 (Organic electrolyte: TEABF_4_/AN)	171	37.1 (device)	^[^ ^91^ ^]^
PaGM	Hydrothermal reaction followed by vacuum drying and heat treatment	891	0.87	4.0 (IL electrolyte: BMIMBF_4_	150	64.7 (device)	^[^ ^39^ ^]^
VASWNTs	Water‐assisted chemical vapor deposition	1300	0.5	4.0 (Organic electrolyte: NEt_4_BF_4_/AN)	80	47.0 (electrode)	^[^ ^92^ ^]^
AHPC	Biodirected surface mesopore engineering followed by KOH activation	3270	0.57	2.0 (Organic electrolyte: TEABF_4_/AN)	120	24 (electrode)	^[^ ^96^ ^]^

^a)^ BNP‐HGH: boron, nitrogen, and phosphorus ternary‐doped holey graphene hydrogel; HGF: holey graphene framework; EM‐CCG: electrolyte‐mediated chemically converted graphene; aMEGO: activated microwave‐expanded graphite oxide; CCNC: collapsed carbon nanocages; h‐graphene: holey graphene; VArGO: vertically aligned reduced graphene oxide (VArGO); MAC‐A: MOF‐derived carbon activated by KOH; ALG‐C: seaweed biopolymer derived amorphous carbons; ac‐Gr/SWCNT: graphene/SWCNT; HPGM: high density porous graphene macroform; PaGM: porosity adjustable graphene monoliths; VASWNTs: vertically aligned SWNTs; AHPC: activated hierarchically porous carbons.

**Figure 10:**
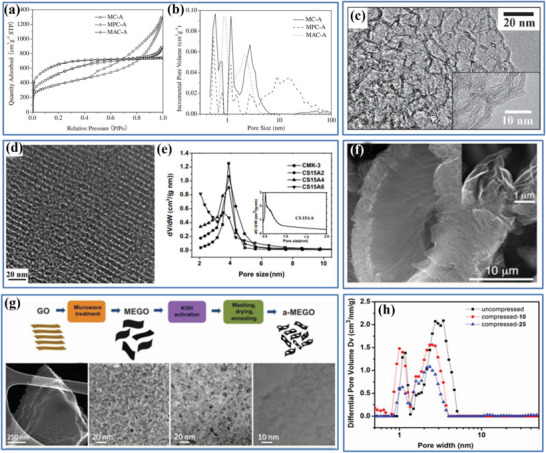
a) Nitrogen adsorption–desorption isotherms and b) DFT pore size distributions of the porous carbons. Reproduced with permission.^[^ ^79d^ ^]^ Copyright 2010, Elsevier. c) A typical TEM image of the as‐prepared CNC annealed at 700 °C. Inset is the corresponding HRTEM image. Reproduced with permission.^[^ ^65b^ ^]^ Copyright 2017, Wiley‐VCH. d) A typical TEM image of CS15A4 material. e) The pore size distribution curves of the porous carbons. Reproduced with permission.^[^ ^80^ ^]^ Copyright 2008, Elsevier. f) A typical SEM image of as‐prepared MEGO by microwave irradiation. Inset is the high magnification SEM image showing the crumpled MEGO sheets. Reproduced with permission.^[^ ^81^ ^]^ Copyright 2010, Elsevier. g) Schematic showing the microwave exfoliation of GO and the following chemical activation of MEGO with KOH that creates pores while retaining high electrical conductivity, and the corresponding SEM and TEM images of the a‐MEGO. Reproduced with permission.^[^ ^82^ ^]^ Copyright 2011, American Association for the Advancement of Science (AAAS). h) The pore size distributions of uncompressed and compressed a‐MEGO‐based electrodes. Reproduced with permission.^[^ ^65a^ ^]^ Copyright 2013, Elsevier.

Carbon activation by either CO_2_ or KOH is the most common strategy to create pores in carbon‐based materials. There are many different precursor materials that have been studied.^[^ ^83^ ^]^ For example, using metal organic framework (MOF‐5) as the template/carbon source and carbon tetrachloride as the additional carbon source, porous carbon (denoted as MAC‐A) with high population of micropores ranging from 0.8 to 2.0 nm and a high SSA of 2222 m^2^ g^−1^ and a *ρ* of 0.93 g cm^−3^ after KOH activation was successfully obtained (**Figure** **10**a,b).^[^ ^79d^ ^]^ Because its major micropore size matches well with the ion size and its high *ρ*, the as‐prepared MAC‐A electrode exhibited a high *C*_vol_ of 252 F cm^−3^ and an *E*_vol‐electrode_ of 8.8 W h L^−1^ in aqueous electrolyte, and a high *C*_vol_ of 145 F cm^−3^ and an *E*_vol‐electrode_ of 29.0 W h L^−1^ in organic electrolyte. This is well consistent with the aforementioned pore size‐ion size matching theory. The KOH activation method has also been applied to hollow/porous CNFs in creating larger mesopores of 3.0–4.5 nm, leading to an improved overall volumetric performance.^[^ ^84^ ^]^ Because there had no studies on the pore arrangement in the carbon/carbon dimension among the pores, it was difficult to provide accurate information on the percentage of electrode material that took part in the double‐layer capacitance. In this regard, porous carbon materials that derived from other carbon precursors through direct carbonization or templating route can provide some new insights. For example, carbonization following by acid etching was used to prepare seaweed biopolymer‐derived amorphous carbons with distributed compact carbon aggregates of multiwalled nanocapsules (100 nm in length) and graphitic shells (15–20 layers).^[^ ^85^ ^]^ In addition to the incorporated oxygen heteroatoms in the porous carbon network contributing pseudocapacitance to the overall electrochemical performance, the high *ρ* of 0.91 g cm^−3^ was another factor that had boosted the *C*_vol_ to 180 F cm^−3^.

Besides the activation approach, mesoporous carbon can also be successfully prepared by an in situ MgO template method with a benzene precursor. As shown in Figure 10c, Hu et al. reported that CNCs with size ranging from 7.0 to 15 nm and a shell thickness of less than 3 nm were obtained by the decomposition of magnesium carbonate at a growth temperature of 700 °C.^[^ ^65b^ ^]^ Although no information on micropores was given in this design, the small nanocage size combined with the thin shell gave rise to a high electrolyte–electrode contact area, leading to an improved electrochemical performance. However, the large hollow size inside the nanocages resulted in low *ρ* and thus relatively poor volumetric performance. In order to address this issue, a thinner shell was prepared on MgO template to collapse the hollow nanocages by capillary force during template removal while maintaining the structural integrity, leading to narrower mesopores (2.0–8.0 nm) in the collapsed nanocages as well as a high *ρ* of 0.76 g cm^−3^ (denoted as hierarchical CNC).^[^ ^86^ ^]^ As a result, the optimal CNC‐based electrode delivered a high *C*_vol_ of 233 F cm^−3^ and an *E*_vol‐stack_ of 73 Wh L^−1^ in IL electrolyte. This strategy provided an insight that even the pore size did not hit microscale (< 2 nm), it was still possible to achieve good volumetric performance by developing very thin carbon and properly arranging the mesopores. Typically, graphene featuring with single atomic layer of graphene/graphite sheet offers the advantage of ensuring theoretically maximum contact area of electrolyte with carbon surface. Nevertheless, the aggregation/restack of graphene flakes tends to form graphite‐like film electrode, which only allows the transport of electrolyte along with the graphene surface rather than penetrating through, limiting the free transport of electrolyte ions and therefore affecting the occurrence of double‐layer capacitance. To this end, the formation of holes/pores with suitable size range on the graphene by the controlled methods (such as Ag‐driven catalytic oxidation of graphitic carbon^[^ ^87^ ^]^ and controlled heating in air^[^ ^65d^ ^]^) is an efficient strategy to build interconnected pores/channels between the aggregated graphene flakes and in theory help the transport of electrolyte ions. This will be further discussed in the following section.

b) Interconnected pores

As discussed above, KOH or CO_2_ activation of bulky carbon often results in random pores which rather independently distribute within the porous carbon matrix. Similarly, when one is using mesoporous carbon, for instance CMK‐3 with highly ordered 2D hexagonal pores, as the starting material, further activation by CO_2_ can help disturb pores and turn them into abundant micropores and interconnected mesopores (3–4 nm), leading to an ultrahigh SSA of 2749 m^−2^ g^−1^ (Figure 10d,e).^[^ ^80^ ^]^ The thus‐made electrode delivered a *C*_vol_ of 54 F cm^−3^ in aqueous electrolyte. The good capacitance performance was mainly attributed to the hierarchically interconnected structure of the well‐balanced micropores and mesopores together with the high SSA. However, to the best of our knowledge, without the presence of proper large channels to ensure an easy transport of electrolyte ions into the carbon, it would be difficult for electrolyte to fully wet and contact the entire surface of micropores and mesopores not to mention the likely poor wettability of electrode with carbon. Therefore, how much of surface was involved as the active carbon in this electrode is still in doubt.

**Figure 11:**
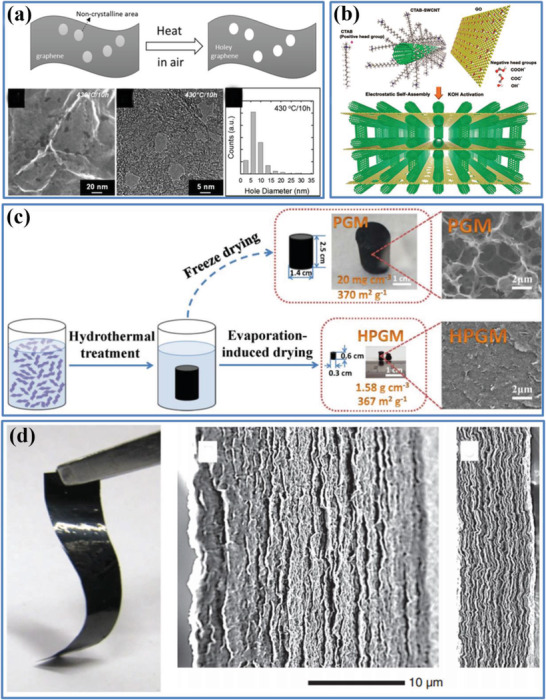
a) Schematic of a scalable synthesis of the h‐Graphene sheets and their corresponding SEM, TEM, and pore size distribution statistic. Reproduced with permission.^[^ ^65d^ ^]^ Copyright 2014, American Chemical Society. b) Schematic for fabricating the ac‐Gr/SWCNT hybrid nanostructure. Reproduced with permission.^[^ ^90^ ^]^ Copyright 2015, American Chemical Society. c) Schematic of the formation of graphene‐based 3D porous macroforms with different drying process and the SEM images of the resultant PGM and HPGM. Reproduced with permission.^[^ ^91^ ^]^ Copyright 2013, Nature Publishing Group. d) A photograph and SEM images of EM‐CCG film. Reproduced with permission.^[^ ^36^ ^]^ Copyright 2013, American Association for the Advancement of Science (AAAS).

Graphite, with parallel stacking of atomic layers of sp^2^ carbon through van der Waals force, possesses an inter‐planar free volume for infusion and transport of small enough ions into every single carbon layer, giving rise to a maximized electrolyte‐carbon contact area. However, its interplanar distance (≈0.335 nm, as presented in **Figure** **11**b) is much smaller than the size of electrolyte ion, leading to a very high ion diffusion resistance. An expansion of the interplanar distance in graphite would give possibility of ion diffusion due to the larger 2D channels between the layers, but the *ρ* decreases accordingly. Microwave irradiation is a useful technique to expand the densely packed graphite layers (Figure 10f).^[^ ^81^ ^]^ The as‐produced crumpled graphitic nanosheets with few‐layer thickness showed a larger interplanar distance of 0.88 nm and higher SSA (463 m^2^ g^−1^) than the pristine graphite oxide, as well as a raised electrical conductivity due to the removal of the oxygen‐containing functional groups. Consequently, it delivered a desirably high volumetric performance. Despite the attempted interplanar space expansion, the 2D nature of the graphitic layers only allows the transport of electrolyte ions through interplanar direction, i.e., the interplanar 2D channels. To this end, a further KOH activation on the expanded graphite to create pores with size of 0.6–5.0 nm on each layers (denoted as a‐MEGO), forming a continuous 3D network of highly curved atom‐thick carbon walls (Figure 10g).^[^ ^82^ ^]^ Benefiting from a very high SSA of 2400 m^2^ g^−1^ and 2D interconnected pores in the graphitic layers, the resulted a‐MEGO‐based electrode delivered a *C*_vol_ of 60 F cm^−3^ and an *E*_vol‐stack_ of 23 Wh L^−1^ in organic electrolyte. Inspired by this work, a further compression on the a‐MEGO film electrode by a standard rolling process collapsed part of the mesopores (≈4.0 nm) to 1.0–2.0 nm micropores (Figure 10h) and increased the *ρ* from 0.34 to 0.75 g cm^−3^, leading to a much higher *C*_vol_ of 110 F cm^−3^ and *E*_vol‐stack_ of 48 Wh L^−1^.^[^ ^83^ ^]^ These results suggest that a proper post‐processing (such as the compression) pressing would effectively densify the porous carbon‐based electrode, and leading to an improvement in double‐layer capacitance.

Instead of expanding the graphite material, an appropriate reassembling of exfoliated graphite, i.e., graphene or GO, with or without holes/pores on the nanolayers, is also able to achieve compact carbon films with well‐balanced *ρ* and pore features. Most commonly, e.g., homogenous GO suspensions in various solvent/surfactant systems are prepared first, and then followed by vacuum filtration and subsequent drying/compression to reassemble the graphene flakes into film electrodes.^[^ ^74^, ^88^, ^89^, ^90^ ^]^ As shown in Figure 11a, prior to the reassembling, the graphene layers shall be carefully heat‐treated in air (e.g., 430 °C for 10 h) to create pores of 2.0–20 nm in sizes (average pore size of 8.0 nm).^[^ ^65d^ ^]^ The following step of vacuum filtration and compression led to a relatively high packing density *ρ* of 1.2 g cm^−3^ for the final graphene film, while the micropores on the layers facilitate the access and penetration of solvent and electrolyte ions. Ascribed to this, a *C*_vol_ of 53 F cm^−3^ and an *E*_vol‐stack_ of 12 Wh L^−1^ can be achieved for h‐graphene‐based electrode in IL electrolyte.^[^ ^65d^ ^]^ In another work, GO suspension was premixed with modified CNT suspension and then filtrated to form CNT/graphene film with a *ρ* of 1.06 g cm^−3^.^[^ ^90^ ^]^ The CNTs were intercalated into the nanoporous graphene layers to build a pillared 3D structure, which increases the accessible surface area and allows for fast ion diffusion (Figure 11b).^[^ ^90^ ^]^ Using IL as the electrolyte, the fabricated EDLC based on these films delivered a high *C*_vol_ of 211 F cm^−3^ and *E*_vol‐electrode_ of 117.2 Wh L^−1^. Besides vacuum filtration method, assembling GO layers into hydrogel is another useful technique to effectively configure graphene layers while achieving reasonably high porosity and pack density. The thus‐obtained hydrogels go through drying/shrinking,^[^ ^90^, ^91^ ^]^ or compression,^[^ ^35^ ^]^ or capillary compression in the presence of a nonvolatile liquid electrolyte,^[^ ^36^ ^]^ to produce “porous and yet dense” carbon films. Drying of the hydrogel led to a “porous and yet dense” graphene macroform (abbreviated as HPGM, Figure 11c), in which the pores are mostly micropores (about 1.1 nm) with a minor amount of mesopores, and the *ρ* is 1.58 g cm^−3^.^[^ ^91^ ^]^ The HPGM‐based EDLC delivered a *C*_vol_ of 171 F cm^−3^ and a high *E*_vol‐stack_ of 37.1 Wh L^−1^ with an organic electrolyte. In addition, by adding ZnCl_2_ as the sacrificial pore former in the hydrogel and applying further annealing after drying, the pore sizes can be precisely tuned over a relatively wide size range.^[^ ^39^ ^]^ Due to a good balance in the level of porosity and *ρ* (a large SSA of 891 m^2^ g^−1^ and a high *ρ* of 0.87 g cm^−3^), the sliced graphene pellet electrode (400 µm thickness) showed a *C*_vol_ of 150 F cm^−3^ in IL electrolyte, corresponding to a high *E*_vol‐electrode_ of 64.7 Wh L^−1^.

While drying is a simple and low‐cost process to extract water from GO hydrogel and collapse the 3D network into compact film, applying compression force onto the hydrogel could more precisely control the pore features and *ρ* of the obtained film. For example, using H_2_O_2_ as an oxidizer and etching agent, holey GO (HGO) was obtained and simultaneously turned into hydrogel, which was then solvated with electrolyte and compressed into holey graphene film.^[^ ^35^ ^]^ In this structure, graphene sheets are interconnected and interlocked into a stable 3D network with large intersheet distance and numerous nanopores on the HGO sheets, leading to a high SSA of 1560 m^2^ g^−1^ and *ρ* of 0.71 g cm^−3^.^[^ ^35^ ^]^ Moreover, the presolvation by electrolyte can fully wet the entire graphene surface, making is an easy access by electrolyte ions. As a result, a high *C*_vol_ of 212 F cm^−3^ and *E*_vol‐stack_ of 49 Wh L^−1^ can be achieved for HGH‐based EDLCs in organic electrolyte.^[^ ^36^ ^]^ These results suggest that the solvation of hydrogel‐based films using a proper solution is able to tailor the level of porosity and stacking of the graphene sheets. Indeed, driven by the selective removal of volatile solvents under capillary compression and the preservation of the nonvolatile solvent in the mixed volatile/nonvolatile solvents–solvated GO hydrogel, a chemically converted graphene‐based film (denoted as EM‐CCG) with high ion‐accessible surface area and well‐mediated *ρ* can be fabricated (Figure 11d).^[^ ^36^ ^]^ By tuning the solvent mix and the volatile/nonvolatile ratio, the *ρ* can be adjusted from 0.13 to 1.49 g cm^−3^, and interestingly, the *C*_vol_ of the obtained film electrode is nearly proportional to *ρ*. Typically, a *C*_vol_ of 255.5 F cm^−3^ in aqueous electrolyte and 261.3 F cm^−3^ in organic electrolyte were obtained for the EM‐CCG film electrode with a pack density of 1.33 g cm^−3^. Accordingly, the EDLC based on EM‐CCG films delivered a high *E*_vol‐stack_ of ≈60 Wh L^−1^, approaching those of lead acid batteries.

**Figure 12:**
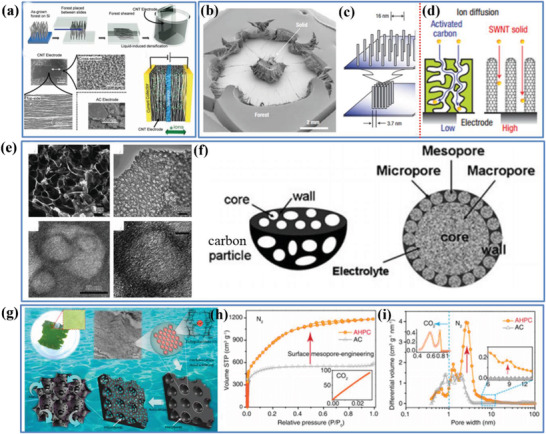
a) Electrode fabrication and characterization of SWCNT electrode. Reproduced with permission.^[^ ^92^ ^]^ Copyright 2010, Wiley‐VCH. b) SEM image of SWCNT‐forest structural collapse from a single drop of liquid. Schematic diagram of c) the collapse of the aligned low‐density as‐grown forest (above) to the highly densely packed SWCNT solid (below), and d) the model comparing the ion diffusion for AC and the SWCNT solid material. Reproduced with permission.^[^ ^94^ ^]^ Copyright 2006, Nature Publishing Group. e) SEM and TEM images of the macroporous cores of the HPGC material. f) Schematic representation of the 3D hierarchical porous texture. Reproduced with permission.^[^ ^95^ ^]^ Copyright 2008, Wiley‐VCH. g) “Egg‐box” model of calcium alginate in brown seaweed and its derived surface mesopore engineering (SME) process for nanoporous carbons. h,i) Pore characterization of seaweed‐derived carbon materials with SME (AHPC) and without SME (AC) treatment. Reproduced with permission.^[^ ^96^ ^]^ Copyright 2015, American Chemical Society.

CNT, as a unique carbon material with extremely high electrical conductivity, possesses continuous inner channel in each of the tube, allowing in theory the occurrence of charge generation and double‐layer capacitance on both outer surface and inner surface. Depending on the wall thickness (single/double/multiwalled), the inner diameter, and wettability of the selected electrolytes on the CNT surface, the transport behavior of electrolyte ions within the inner channels varies from one another in terms of the easiness and completeness. Simple accumulation of the CNTs into a compact film is the most straightforward process of using this material as the electrode for EDLC. First, however the poor contact/compatibility among the CNTs and between the CNT film and the current collector often leads to a much lower electrical conductivity. This can be addressed by adopting high purity single‐walled CNT (SWCNT) or enhancing the interaction among CNTs through electro‐statistic force.^[^ ^92^, ^93^ ^]^ For example, the SWCNTs (>99.98% carbon purity) were synthesized by water‐assisted chemical vapor deposition to achieve a high SSA (1300 m^2^ g^−1^) and high electrical conductivity. They were then assembled into thin sheets by shearing between glass slides, followed by prewetting in selected electrolyte to achieve a densely packed film (0.5 g cm^−3^) but accessible electrolyte (**Figure** **12**a).^[^ ^92^ ^]^ Ascribed to these merits, the fabricated EDLC based on SWCNT‐films yielded an *E*_vol‐electrode_ of 47 Wh L^−1^ in organic electrolyte. Second, the CNTs in the film are randomly oriented, thus the diffusion direction of electrolyte ions within the CNTs can contradict the “charge–discharge direction” in the device, slowing down the ion diffusion. Iijima et al. reported the formation of highly packed SWCNTs with length in millimeter‐scales and aligned orientation being perpendicular to the current collector, through densification of the as‐grown SWCNT arrays by liquid‐mediated “zipping” effect (Figure 12b–d).^[^ ^94^ ^]^ The liquid surface tension and the van der Waals interactions were utilized to narrow the spacing of the adjacent CNTs from 16 nm down to 3.7 nm, without damaging their structure integrity, accompanying an increased pack density from 0.03 to 0.78 g cm^−3^. Therefore, the advantages of SWCNT are maintained, including a large SSA, high electrical conductivity, and superior flexibility; and what is more critical, the electrolyte ions can easily diffuse in the nanochannels between these aligned SWCNTs. Compared with those with random nanopores of AC, a high ion accessibility is ensured. When used as a binder‐free film electrode for EDLC, a better volumetric performance was achieved, compared with those as‐grown SWCNT arrays and AC film electrodes.

c) Hybrid pores

Compared with the assembling of graphene sheets or CNTs into “porous and yet dense” films with interconnected pores, which have the risk of being poor or unstable in connection among graphene sheets or CNTs, the construction of hybrid pores (i.e., a combination of micropores and/or mesopores and/or macropores) in bulky carbon materials has the primary advantages of being higher structural integrity and stability. As such, macropores can act as a buffering zone to accommodate electrolyte, mesopores provide pathway for low‐resistant transport of electrolyte ion to wet and contact with carbon surface, and micropores of 0.8–2.0 nm in sizes provide a large surface area for charge generation and accumulation. To meet the “porous and yet dense” requirement for optimal volumetric performance, one has to configure the macropores, mesopores, and micropores properly in order to fully realize their functions. Porous carbons with hybrid pores have been successfully prepared by several different techniques, such as templating, chemical activation of structured carbon, and conversion from natural resources/precursors with desired pore features. For example, a hierarchically porous graphitic carbon (HPGC, Figure 12e,f) was prepared by forming an inorganic hydroxide/phenolic resin composite, followed by carbonization and template removal.^[^ ^95^ ^]^ The resultant carbon comprises macroporous cores (60–100 nm), mesoporous walls (5–50 nm) and micropores (<1 nm and 1–2 nm), with a total SSA of 970 m^2^ g^−1^, a total pore volume of 0.69 cm^3^ g^−1^, a micropore volume of 0.3 cm^3^ g^−1^, and an average pore size of 2.85 nm. Compared with commercially available AC and ordered porous carbon, the porous HPGC electrode possesses better volumetric performance.

As has been discussed in Section 3.1.2, those micropores of <1.0 nm in sizes may not largely contribute to the double‐layer capacitance through desolvation of ions, due to the large ion diffusion resistance. A significant population of mesopores and macropores of large pore sizes may render the carbon a relatively low *ρ*. Consequently, both two limitations will lower the volumetric performance of porous carbon‐based electrodes. These issues are also found in 3D hierarchical porous carbon (3D HPC, Figure 12e,f) that was prepared by chemical activation of polypyrrole macrosheets.^[^ ^97^ ^]^ Although a high SSA of 2870 m^2^ g^−1^ and a high *C*_wt_ of 318.2 F g^−1^ were achieved for the 3D HPC‐based electrode, its *C*_vol_ and *E*_vol_ are not satisfying, due to the very wide size range (1.7–300 nm) and resultant low *ρ*. In this regard, the use of appropriate natural resources with limited population of macropores as the carbon precursor (such as seaweed) can help achieve properly arranged hybrid pores, which could give rise to excellent volumetric performance. Through carbonization and activation by KOH, seaweed can be converted to “egg‐box” microcrystalline carbons with hierarchically porous structure (denoted as AHPCs, Figure 12g–i).^[^ ^96^ ^]^ The obvious difference between the AHPC and aforementioned carbon with hybrid pores is its narrower pore size distribution of 0.5–8.0 nm (mainly in the range of 2.0–4.0 nm), with only few macropores (Figure 12e). Moreover, the ratio of mesopores‐induced surface area to micropores‐induced one is much higher compared with commercial AC (20.9 for the AHPC vs 0.85 for AC), suggesting a dense pore feature (a *ρ* of 0.57 g cm^−3^ and an SSA of 3270 m^2^ g^−1^) with high ion transport capability. As a result, the AHPC‐based EDLC gave a *C*_vol_ of 120 F cm^−3^ and an *E*_vol‐electrode_ of 24 Wh L^−1^ in organic electrolyte. This work further proves that, in design of the hybrid pores, the macropores and large‐size mesopores can contribute to the *C*_wt_, but not much benefit to the volumetric performance. Indeed, although hybrid pores could be formed in the carbon structures that were prepared from some natural resources (such as ant powder and silkworm),^[^ ^98^ ^]^ the relatively large amount of interconnected macropores of few hundred nanometers in sizes renders a low *ρ* (0.2 g cm^−3^ for the silkworm‐derived carbon). Therefore, low volumetric performance values are the trade‐off for the easy electrolyte transport in such macropores.

d) Aligned pores

**Figure 13:**
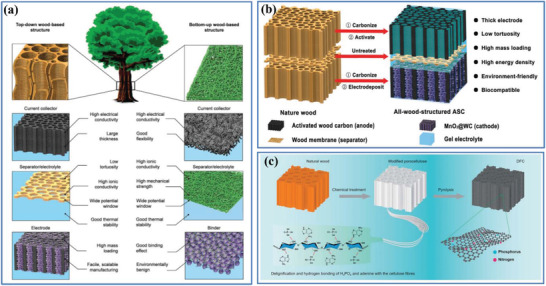
a) Overall picture of wood‐based structures for electrochemical energy storage application. Reproduced with permission.^[^ ^78b^ ^]^ Copyright 2021, Wiley‐VCH. b) Graphical illustration of the design concept and construction process of the all‐wood‐structured supercapacitor. Reproduced with permission.^[^ ^77a^ ^]^ Copyright 2017, Royal Society of Chemistry. c) Schematic illustration of the DFCs fabricated from a modified porocellulose, obtained via a one‐pot chemical treatment of natural wood. Reproduced with permission.^[^ ^100a^ ^]^ Copyright 2020, Royal Society of Chemistry.

A “porous and yet dense” carbon electrode featured with aligned pores shows a low tortuosity, in which anisotropic pores are preferentially oriented in the primary transport direction, contributing to faster transport of ions and electrons by reducing the diffusion distance. Recently, several prominent studies have reported that minimizing/lowering the tortuosity of porous carbon electrodes with a straight and aligned channel (pore) can lead to an improved volumetric capacitance, while maintaining a good rate capability.^[^ ^77^, ^78^, ^99^ ^]^ Inspired by some of the natural wood materials with aligned channels along the growth direction (**Figure** **13**a),^[^ ^78b^ ^]^ Hu et al. obtained a high‐conductive, lightweight, and low‐tortuosity 3D wood‐carbon monolith by carbonizing from natural wood, which can not only directly act as active materials for negative electrode (AWC) and a membrane separator, but also as an ideal current collector to grow MnO_2_ nanosheets for positive electrode (MnO_2_@WC).^[^ ^77a^ ^]^ Benefiting from the three components all show unique anisotropic structure with numerous aligned channels/pores along the direction of ions and electrons transport ensuring a low tortuosity, the as‐assembled asymmetric EC delivered remarkably high energy and power densities, as well as an outstanding rate performance (Figure 13b).^[^ ^77a^ ^]^ Following this work, on the one hand, various pseudocapacitive materials such as Co(OH)_2_@WC^[^ ^99a^ ^]^ and PEDOT@FeOOH@WC^[^ ^99b^ ^]^ have been grown inside or impregnated into the aligned channels/pores in order to obtain high‐performance composite electrodes for ECs.

On the other hand, doping of additional heteroatoms (such as N, P, and S) to functionalize the low‐tortuosity 3D wood‐carbon monolith can not only improve its electronic conductivity that further facilitates to faster electron transport, but also introduces pseudocapacitance without adding extra materials.^[^ ^100^ ^]^ For example, Huang et al. developed a structure‐engineered and dual‐heteroatom functionalized 3D wood‐carbon slice with directional ion shortcuts, efficient electron conduction, and sufficient active sites as the ultrathick electrode for high‐volumetric‐capacitance ECs.^[^ ^100a^ ^]^ This unique combination of structural and functional design is motivated by low‐tortuosity architectures and the abundant hydrogen bonds, which can be achieved by pyrolyzing the as‐modified porous cellulose following a one‐pot chemical treatment of natural wood. Similarly, the 3D heteroatom‐doped, low‐tortuosity wood‐carbon electrodes with plenty of well‐connected open channels have also been reported by Zhi et al., showing improved volumetric capacitance, while maintaining good rate performance.^[^ ^100b^ ^]^

## 4 Porous Pseudocapacitive Electrodes for High‐Volumetric Performance

### 4.1 Nanoporous/Nanostructuring Pseudocapacitive Materials

As has been discussed in Section 2.1.2, PCs store charges via chemically fast and reversible Faradaic reactions at the surface/near‐surface of the pseudocapacitive materials (e.g., transition metal oxides or conductive polymers). To this end, advanced pseudocapacitive electrodes shall possess a porous/nanoporous structure, which not only benefits the electrolyte penetrating into active materials, but also largely speeds up the ion transport across the entire surface. Indeed, looking back to the discovery of pseudocapacitance in RuO_2_, the nanoporous/nanostructuring enhanced the interfacial area between the electrolyte and the RuO_2_, and provided more reactive sites accessible for charge transfer reaction, giving rise to much improved overall electrochemical performance.^[^ ^101a^ ^]^ Simultaneously, the slow reaction rate could be largely raised to decrease the charging/discharging times to ≈10 s by absorbing a higher volume of electrolyte into the porous electrode. In another example, the pseudocapacitive Li^+^ intercalation kinetics in the nanoporous/nanostructured T‐Nb_2_O_5_ with high surface area and numerous mesoporous was reported by Brezesinski et al. in 2010.^[^ ^101b^ ^]^ The mesoporous film electrodes are assemblies of nanostructured materials that can provide short solid‐state diffusion distances as well as electronic conduction through interconnected grains and high porosity for electrolyte accessibility. Moreover, highly porous metal‐organic frameworks (MOFs) can offer intrinsically pseudocapacitive charge storage, due to their crystalline structures with highly tunable and large porosity as well as good electronic and fast ionic conduction; thus, they have been demonstrated as high‐performance PC electrodes.^[^ ^101c–e^ ^]^

It should be pointed that these discussions in association with pseudocapacitive behavior only be applied to an individual electrode, while the pseudocapacitive materials are usually used as the positive electrodes or negative electrodes to assemble asymmetric EC device (Figure 3d). The other type of asymmetric EC device is HC, in which one electrode stores charge based on the capacitive mechanism (carbon materials or pseudocapacitive materials) while the other electrode stores charge by a battery‐type Faradaic process (as we shown in Figure 2d). In this connection, on the one hand, the structural requirements of the battery‐type electrode for HC are similar to PC electrode. On the other hand, as the investigation into battery‐type materials is still at relatively early stage and some of the corresponding charge‐storage mechanisms are not very clear, only a few works about “porous and yet dense” HC electrodes have been reported. Thus, we will not review the recent progress on the rational design and development of “porous and yet dense” battery‐type electrodes for high‐volumetric‐performance HCs.

### 4.2 Porous/Nanoporous Pseudocapacitive Electrodes for High‐Volumetric‐Performance PCs

Depending on the materials nature and the reaction process involved, transitional metal oxides,^[^ ^101f^ ^]^ conductive polymers,^[^ ^102^ ^]^ and MXenes^[^ ^103a^ ^]^ have been widely investigated as electrode materials for PCs. It should be noted that the pseudocapacitive behavior of these materials largely relies on their key structure parameters (such as particle sizes or film thickness, and amorphous or crystalline forms).^[^ ^103b–e^ ^]^ For example, both amorphous and crystalline forms of MnO_2_ have been studied for high‐rate energy storage. It has been reported that charge storage in amorphous MnO_2_ occurs mainly on the surface, while crystalline MnO_2_ possesses an additional capacitive contribution from bulk ion intercalation.^[^ ^103e^ ^]^ In addition, due to the poor electrical conductivity, some of them were structured with porous carbon materials to facilitate the charge conduction. In the following part of this section, we will look into the pore designs and engineering strategies in PC electrodes using some typical pseudocapacitive materials (MnO_2_, polyaniline [PANI], and MXenes), as examples of elaborations based on material nature, especially those in the recent years, for improved volumetric performance (**Table** **3**).

a) Porous/nanoporous transitional metal oxides

**Figure 14:**
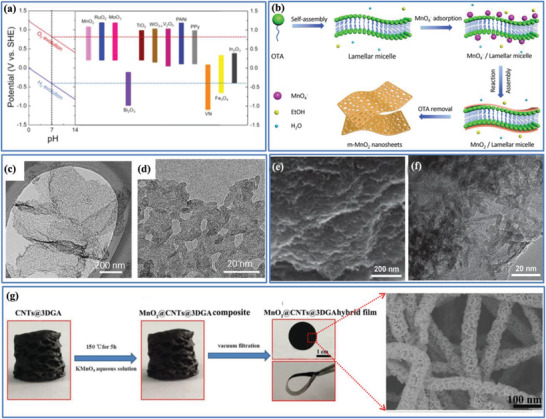
a) Schematic representation of the electrochemical stability range of water and potential windows versus SHE for different PC materials in an aqueous electrolyte. Reproduced with permission.^[^ ^21^ ^]^ Copyright 2018, American Chemical Society. b) Schematic showing the synthesis of 2D m‐MnO_2_ nanosheets. c,d) TEM images of 2D m‐MnO_2_ nanosheets. Reproduced with permission.^[^ ^104^ ^]^ Copyright 2019, Elsevier. e,f) SEM and TEM images of porous yet densely stacked MnO_2_@graphene composites. Reproduced with permission.^[^ ^105^ ^]^ Copyright 2016, Wiley‐VCH. g) Illustration of preparing the MnO_2_@CNTs@3DGA hybrid film. The inset is the SEM image of MnO_2_@CNTs @3DGA hybrid. Reproduced with permission.^[^ ^6a^ ^]^ Copyright 2017, Wiley‐VCH.

A number of transitional metal oxides (such as MnO_2_, V_2_O_5_, MoS_2_, MoO_3_, WO_3_, VN, TiO_2_, and Nb_2_O_5_, **Figure** **14**a) are electrochemically active as the PC electrodes, for which the pore features can be precisely controlled. Our groups in Singapore have conducted broad explorations in designing novel nanostructures and PC devices, some of which were focused on pore creation and engineering, to achieve high volumetric performance.^[^ ^25^, ^106^ ^]^ Taking MnO_2_ as an example, due to its relatively low cost, natural abundance, and high theoretical capacitance, various strategies have been developed to fabricate porous MnO_2_ structures for improving their electrochemical performance. While many works have been focusing on the improvement of *C*_wt_,^[^ ^107^ ^]^ the typical ones on the *C*_vol_ are summarized here to demonstrate the engineering of pores, and how they affect the ultimate performance. As among the most common forms, nanosheets/nanoflakes of MnO_2_ were synthesized through a bottom‐up self‐assembly route (Figure 14b).^[^ ^104^ ^]^ As shown in Figure 14c,d, MnO_2_ nanosheets with a thickness of 10 nm showed rather uniformly distributed mesopores of 5.0–15 nm and an SSA of 128 m^2^ g^−1^ (denoted *m*‐MnO_2_). As a proof‐of‐concept for potential application, an all‐solid‐state asymmetric micro‐supercapacitor based on the m‐MnO_2_ nanosheets as the positive electrode and porous VN nanosheets as the negative electrode delivered a *C*_vol_ of 38.8 F cm^−3^ and an *E*_vol‐electrode_ of 21.6 mWh cm^−3^, in which the in‐plane mesopores play an important role to facilitate the transport of electrolyte ions. In another work, a highly concentrated ink containing porous *δ*‐MnO_2_ nanosheets with an average lateral size of 89 nm and around 1 nm thickness was prepared, and then inkjet printed on the substrate together with poly(3,4‐ethylenedioxythiophene) (PEDOT):polystyrene sulfonate (PSS) to fabricate an all‐solid‐state symmetric MSC,^[^ ^108^ ^]^ where the inject printing is a useful method to obtain “porous and yet dense” electrode based on MnO_2_ wrinkled nanosheets. However, the as‐fabricated device exhibited a rather low *C*_vol_ of 2.4 F cm^−3^ and *E*_vol‐electrode_ of 0.18 mWh cm^−3^ because the intersheet space may be too small for smooth and fast diffusion of electrolyte ions due to the too dense packing. Therefore, the balance in level of porosity (such as pore size, pore connectivity, etc.) and *ρ* is a key point that largely influences the electrolyte/ions transport as well as the contact area between active materials and electrolyte. Generally, *α*‐MnO_2_ shows a tunnel‐typed microstructure, which consists of a series of [2 × 2] and [1 × 1] tunnels formed by double chains of edge‐sharing MnO_6_ octahedra cross‐linked by sharing corners, while *δ*‐MnO_2_ possesses a layered crystalline structure built up from layers of edge‐sharing MnO_6_ octahedra with a 0.7 nm interlayer spacing.^[^ ^106^, ^109^ ^]^ The unique layered crystalline structure of *δ*‐MnO_2_ offers a higher ions accessibility and lower charge transfer resistance than the tunnel‐structured *α*‐MnO_2_.^[^ ^106^, ^109^ ^]^ Furthermore, the incorporation of *δ*‐MnO_2_ nanocrystals on nanoporous Au patterns via pulse electrodeposition forms 3D nanoporous *δ*‐MnO_2_ composites. The as‐obtained microelectrode exhibited a high *C*_vol_ of 922 F cm^−3^ and a maximum *E*_vol‐electrode_ of 24.3 mWh cm^−3^ in aqueous electrolyte.

**Table 3:** Summary of “porous and yet dense” electrodes based on pseudocapacitive materials discussed in this review

Electrode materials^a)^	Preparation methods for electrode	Specific surface area [SSA, m^2^ g^−1^]	Packing density [g cm^−3^]	Working voltage window [V]	Maximal volumetric capacitance [*C*_vol_, F cm^−3^]	Maximal volumetric energy density [*E*_vol_, W h L^−1^]	Refs.
m‐MnO_2_//PVN	Supramolecular bottom‐up self‐assembly strategy	128	NA	2.0 (Symmetric, SiO_2_‐LiTFSI gel electrolyte)	38.8	21.6 (electrode)	^[^ ^94^ ^]^
NP Au/MnO_2_	Pulse electrodeposition	NA	NA	0.8 (Symmetric, Na_2_SO_4_ gel electrolyte)	922	24.3 (electrode)	^[^ ^109a^ ^]^
G‐MnO_2_/DGS	Modified Hummers method followed by hydrothermal treatment	74	NA	1.8 (Asymmetric, Na_2_SO_4_ gel electrolyte)	366	54.4 (electrode)	^[^ ^105^ ^]^
MnO_2_@CNTs@3DGA/ Ppy@CNTs@3DGA	Hydrothermal method and CVD followed by redox reaction	NA	NA	1.8 (Asymmetric, Na_2_SO_4_ gel electrolyte)	8.56	3.85 (electrode)	^[^ ^6^ ^]^
PANI‐CCG/ PANI‐CCG	Vacuum filtration of CCG dispersion followed by in situ chemical polymerization	NA	1.25	0.9 (symmetric, H_2_SO_4_ liquid electrolyte)	572	15.8 (electrode)	^[^ ^37^ ^]^
NFHG/PANI	Hydrothermal treatment followed by in situ chemical polymerization	196	1.43	1.0 (three‐electrode, Na_2_SO_4_ liquid electrolyte)	828	NA	^[^ ^126^ ^]^
PHCFs@PANI/ PHCFs@PANI	Multiple dip‐coating technique and porosity engineering followed by electrodeposition	NA	NA	0.8 (symmetric, H_2_SO_4_ gel electrolyte)	17.4	1.55 (electrode)	^[^ ^123^ ^]^
K^+^ intercalated Ti_3_C_2_T*_x_* paper	Intercalation of K^+^ ion between the Ti_3_C_2_T*_x_* layers	98	NA	0.5 (three‐electrode, KOH liquid electrolyte)	340	18.5 (electrode)	^[^ ^127a^ ^]^
Ti_3_C_2_T*_x_* clay film	Rolling the prepared clay‐like paste	NA	3.6–3.8	0.55 (three‐electrode, H_2_SO_4_ liquid electrolyte)	900	NA	^[^ ^65g^ ^]^
3d‐Ti_3_C_2_T*_x_*‐film	Vacuum filtration and rapid freezing	45.1	3.65	1.0 (symmetric, H_2_SO_4_ liquid electrolyte)	931	32.2 (electrode)	^[^ ^131^ ^]^
MP‐MX1.5	Incorporating Fe(OH)_3_ nanoparticles into MXene film followed by calcination	56	3.3	0.8 (three‐electrode, H_2_SO_4_ liquid electrolyte)	1142	20.7 (electrode)	^[^ ^137^ ^]^
MnO_2_@MXene composite film	Solution processing of hybrid inks followed by vacuum filtration	NA	NA	0.8 (symmetric, LiCl gel electrolyte)	1025	56.9 (electrode)	^[^ ^140^ ^]^
CNT@MXene	Alternating filtration	NA	2.9	0.9 (three‐electrode, Mg_2_SO_4_ liquid electrolyte)	390	NA	^[^ ^142^ ^]^
rGO@MXene	Electrostatic self‐assembly followed by vacuum filtration	68.1	3.1	1.0 (symmetric, KOH liquid electrolyte)	1043	32.6 (electrode)	^[^ ^143^ ^]^

^a)^ m‐MnO_2_: mesoporous MnO_2_, PVN: porous VN; NP Au/MnO_2_: 3D bicontinuous nanoporous Au/MnO_2_ composites; G‐MnO_2_: MnO_2_‐intercalated graphite oxide, DGS: densely stacked graphene; CNTs@3DGA: CNTs@3D graphene aerogel; NFHG/PANI: N and F co‐doped holey graphene/PANI; PHCFs@PANI: porous, hollow, and conductive composite fibers@PANI; MP‐MX1.5: modified nanoporous MXene film.

On the other hand, to address the well‐known issue of relatively poor electrical conductivity of transitional metal oxides, a common strategy is to design porous/nanoporous hybrids by combining metal oxides with electrically conductive materials such as carbon (e.g., graphene^[^ ^110^ ^]^ and CNT^[^ ^111^ ^]^), conductive polymer (e.g., PPy^[^ ^112^ ^]^ and PANI^[^ ^113^ ^]^), and metal (e.g., Ni^[^ ^114^ ^]^). More importantly, the nanohybrids of conductive materials were also widely selected and engineered.^[^ ^115^ ^]^ For example, a “porous and yet densely” stacked MnO_2_@graphene composite (the ratio of MnO_2_ is 74 wt%) was prepared via a solution route.^[^ ^105^ ^]^ As shown in Figure 14e,f, the composites feature in numerous MnO_2_ nanorods (6.0–7.0 nm in diameter and 15–20 nm in length) that uniformly and separately intercalate into graphene sheets with strong interaction to form tightly packed and yet porous structure with considerable amount of micropores (2.0–5.0 nm). Consequently, the asymmetric EC based on MnO_2_@graphene composites as the positive electrode and graphene as the negative electrode delivered a high *C*_vol_ of 366 F cm^−3^ and a maximum *E*_vol‐electrode_ of 54.4 mWh cm^−3^ in aqueous electrolytes. In fact, the simple assembling of graphene and MnO_2_ shows an instability issue. To this end, direct deposition or growth of porous MnO_2_ nanosheets on a stable scaffold offers a higher structural integrity. In one of the previous works, a hierarchically porous carbon hybrid was first fabricated by growing 1D CNTs on 3D graphene aerogel (CNTs@3DGA), which was then acted as a promising scaffold for depositing MnO_2_ nanosheets to obtain MnO_2_@CNTs@3DGA composites (the mass loading of MnO_2_ up to 7.5 mg cm^−2^) (Figure 14g).^[^ ^6a^ ^]^ The optimized all‐solid‐state asymmetric EC based on MnO_2_@CNTs@3DGA and Ppy@CNTs@3DGA film electrodes exhibited a high *E*_vol‐electrode_ of 3.85 mW h cm^−3^ and superior long‐term cycle stability with 84.6% retention after 20 000 cycles. Such excellent overall performance is attributed to the “porous and yet dense” film electrodes possessing a high structural integrity between the MnO_2_ materials and the 3D CNTs@3DGA network.

**Figure 15:**
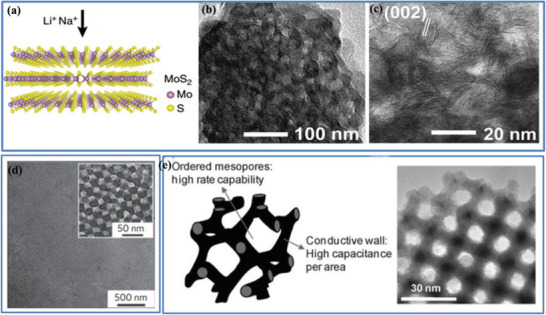
a) Schematic showing the crystal structure of MoS_2_. TEM images of the mp‐MoS_2_ b) before and c) after cycling. Reproduced with permission.^[^ ^116^ ^]^ Copyright 2016, Wiley‐VCH. d) TEM image of mesoporous *α*‐MoO_3_ with highly oriented crystalline walls. Reproduced with permission.^[^ ^117^ ^]^ Copyright 2010, Nature Publishing Group. e) Schematic explanation of the high‐performance mesoporous WO_3−_ *_x_* electrode, and TEM image of mesoporous WO_3−_ *_x_*. Reproduced with permission.^[^ ^118^ ^]^ Copyright 2011, Royal Society of Chemistry.

Besides MnO_2_, other pseudocapacitive materials (such as WO_3_, MoO_3_, V_2_O_5_, MoS_2_, VN, TiO_2_, and Nb_2_O_5_) have also been designed with porous/nanoporous structures with improved overall electrochemical performance for PCs. The great potential of extrinsic pseudocapacitance in nanostructured and exfoliated transition metal dichalcogenides has been demonstrated in many recent studies. Among them, MoS_2_ holds great promise as a negative material for asymmetric EC, due to its 2D layered structure (analogous to graphene) and the tunable change in electronic properties. Tolbert et al. reported that mesoporous MoS_2_ can be utilized as a pseudocapacitive energy storage material with a high volumetric performance and much increased rate capability (**Figure** **15**a,b), which can be attributed to the fast electrochemical kinetics correlated with the ordered porous structure and with an iso‐oriented crystal structure.^[^ ^116^ ^]^ Similarly, the pseudocapacitive charge‐storage properties of ordered mesoporous *α*‐MoO_3_‐ (Figure 15c) and WO_3−_ *_x_*‐based (Figure 15d) film electrodes are superior to their nonporous counterparts,^[^ ^117^, ^118^ ^]^ which can be attributed to the large SSA, high electrical conductivity, and high *ρ* .

Those studies shown in above‐mentioned works have indeed proven that the nanoporous/nanostructuring, in the form of nanosheets or nanowires of pseudocapacitive materials, can not only increase the interfacial area between the electrolyte and active materials and provide more reactive sites accessible for charge transfer reaction, but also decrease the ion diffusion distance, leading to an improved volumetric capacitance and rate capability. More important, to combine these nanoporous/nanostructuring pseudocapacitive materials with highly conductive carbon material overcomes the limitation that modest levels of conductivity constrain the charge transport properties and leads to a low power density. Although significant progress has been achieved on the rational design of advanced transitional metal oxides, it is still essential to develop more pseudocapacitive materials with “porous and yet dense” structure (such as conductive polymers and MXenes) in fabrication of asymmetric ECs with enhanced volumetric performance.

b) Conductive polymers

Conducting polymers, a class of pseudocapacitive materials established by Shirakawa et al.,^[^ ^119^ ^]^ were widely investigated as a class of materials for PCs since the late 1980s,^[^ ^37^, ^120^, ^121^, ^122^, ^123^, ^124^ ^]^ which store charge via the mechanism of highly eversible electrochemical doping–dedoping. This process involves two parts, i.e., ions are transferred into the polymer backbone (positively charged polymers, termed as p‐doping) when oxidation occurs, and ions are released from this backbone into the electrolyte when reduction takes place (negatively charged polymers, termed as n‐doping). These redox reactions in the conducting polymers come about throughout their entire bulk, not just on the surface/near‐surface. Considering this, one would conclude that porous conducting polymers with high SSA and controlled pore features shall deliver a higher electrochemical performance than their nonporous counterparts. Nonetheless, although an excellent capacitance can be achieved for most conducting polymers, the major challenges are their swelling and shrinking during the intercalating/deintercalating process, resulting in a short PC lifetime. Specifically, the cycling degradation issue is resulted from the volumetric changes or mechanical forces of conducting polymers during the charging/discharging process. To this end, nanoporous/nanostructured conducting polymer could address this issue by offering a relatively short diffusion length to increase the utilization of electrode materials.

Up to date, various conductive polymers including PANI, PPy, polythiophene (PTh), and PEDOT and their corresponding derivatives have shown the pseudocapacitive charge storage behaviors.^[^ ^122^ ^]^ Among them, the PANI possessing the highest theoretical *C*_wt_ of 750 F g^−1^ is the most commonly used positive materials for PCs; thus, we will take PANI as an example to emphasize the importance of pore‐engineering on its ultimate electrochemical performance in the following discussions.

**Figure 16:**
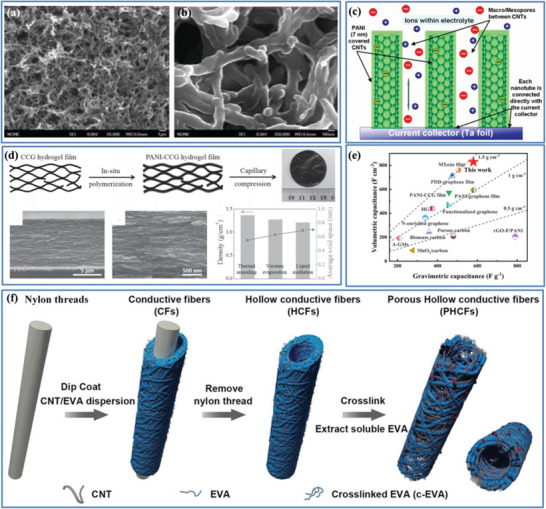
a,b) SEM images of the PANI film deposited by pulse galvanostatic method in a reverse micelle electrolyte. Reproduced with permission.^[^ ^125^ ^]^ Copyright 2011, Royal Society of Chemistry. c) Schematic representation of the microstructure and energy storage characteristics of the PANI/CNT composite. Reproduced with permission.^[^ ^124^ ^]^ Copyright 2008, Elsevier. d) Fabrication and characterization of compact PANI‐CCG films. Reproduced with permission.^[^ ^37^ ^]^ Copyright 2016, Wiley‐VCH. e) Comparison of the volumetric and gravimetric capacitances of the NFHG/PANI film with other reported materials. Reproduced with permission.^[^^121c]^ Copyright 2021, Springer. f) Schematic illustration of fabricating PHCFs. Reproduced with permission.^[^ ^123^ ^]^ Copyright 2018, Royal Society of Chemistry.

Among the most convenient techniques of using PANI are to produce powders and thin film‐on‐substrate through chemical oxidation polymerization or electrochemical polymerization deposition. Moreover, the as‐obtained PANI powders can be casted into thin films, in which separated pores could be created through adding porogens (benzoyl peroxide (BPO), azoisobutyronitrile (AIBN), ammonium bicarbonate ([AB), etc.) into the homogenous PANI suspension.^[^ ^120^ ^]^ The resultant pore features were affected by the decomposition kinetics of the porogens and the releasing of produced gas, giving rise to pore sizes within 30–150 nm range. In addition, the surface roughness of the thin film was also increased by about two times, contributing to wet the PANI surface by the electrolyte. For example, the optimized thin‐film electrode based on porous PANI (**Figure** **16**a,b) was shown to deliver a superior gravimetric and volumetric capacitance than solid PANI electrode.^[^ ^125^ ^]^ Such improved overall performance can be ascribed to the two aspects: i) the “porous and yet dense enough” structure that comprises the nanofibrillar PANI with diameter of 100–200 nm facilitates the rapid ionic transport within the pores to offer access for the ions to reach the surface of active materials; ii) the interconnected pores/channels among the nanofibrillar PANI have a size of a few hundred nanometers, which provide pathway for electrolyte/ions transport for easy redox reaction. Although it is intrinsically electric‐conductive, the major drawbacks of conductive polymers are their noncomparable conductivity to carbon materials and relatively poor structural stability. The integrity of conductive polymers with carbon or metal materials to form conductive polymer composites is an efficient strategy to address the mentioned issues (Figure 16c).^[^ ^124^ ^]^ For example, Li et al. reported that a series of “porous and yet densely” compacted PANI‐CCG films were prepared by controlled compression of PANI‐CCG hydrogels, in which the high pore connectivity can not only guarantee rapid ion transport and well‐maintained nanostructured PANI, but also pay a critical role to fast kinetics of Faradaic charge‐storage processes (Figure 16d).^[^ ^37^ ^]^ Consequently, the optimized PANI‐CCG hydrogel film was indeed able to provide a combination of high‐rate capability, high *C*_vol_ of 572 F cm^−3^, and long cycling life. Similarly, a free‐standing binder‐free N and F co‐doped holey graphene/PANI (NFHG/PANI) composite film with a high *ρ* of 1.43 g cm^−3^ was fabricated by Liu et al., which possessed a superior *C*_vol_ of 800 F cm^−3^ (Figure 16e).^[^ ^121c^ ^]^ Such high volumetric performance is of great importance for the application of NFHG/PANI composite film in miniaturized portable energy storage devices.

Carbon‐based materials are durable and mechanically strong enough to be processed and treated by various procedures in order to create micro‐/meso‐/macropores and their combinations. However, it is challenging to precisely engineer the pore size and pore connectivity of the conductive polymer structures due to their mechanical disadvantage and the relatively monotonous synthesis methods, especially to achieve micropores and mesopores with sizes of less than 5 nm. The relatively large pore size and poor pore connectivity may result in low volumetric performance. In fact, despite the aforementioned works provided useful strategies and insights to construct separated or interconnected or hybrid pores in the structures of conductive polymers and thus demonstrated desirable gravimetric performance, most of them had delivered rather low volumetric performance, limiting their wide application in next‐generation wearable electronics. To this end, a high‐volumetric‐performance fiber‐shaped EC (FSEC) was fabricated based on a new class of porous, hollow, and conductive polymer composite fibers (PHCFs) with a multilayer structure.^[^ ^123^ ^]^ Utilizing the 3D porous nanoarchitectures with hybrid pores including micropores, mesopores, and macropores as substrate (Figure 16e), the PANI can be introduced into the structure via a multiple dip‐coating technique and porosity engineering. Guided by the hybrid pores in the PHCFs, the electrolyte ions have full access to the surface of loaded PANI, leading to an *E*_vol‐stack_ of 1.55 mWh cm^−3^ at a power density of 12 mW cm^−3^, values far superior to those of most recently reported FSECs.

c) MXenes

**Figure 17:**
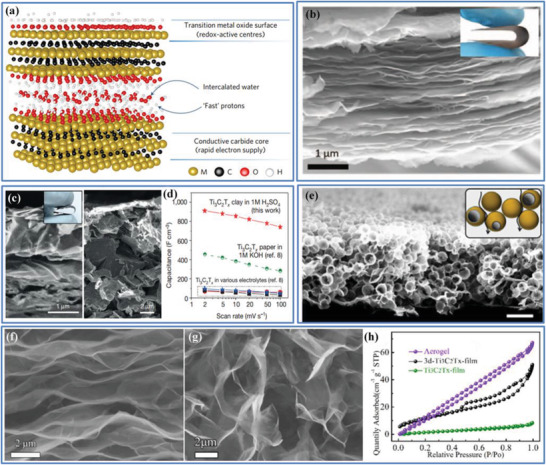
a) Schematic illustration of MXene structure. Reproduced with permission.^[^ ^128^ ^]^ Copyright 2017, Nature Publishing Group. b) SEM image of a binder‐free intercalated Ti_3_C_2_T*_x_* film electrode. Reproduced with permission.^[^ ^129^ ^]^ Copyright 2019, Elsevier. c) Cross‐sectional SEM image of the 4 and 20 µm clay‐like additive‐free Ti_3_C_2_T*_x_* films. d) Comparison of rate performance of this work and previously reported MXene films. Reproduced with permission.^[^ ^65g^ ^]^ Copyright 2014, Nature Publishing Group. e) Cross‐sectional SEM image of macroporous‐templated Ti_3_C_2_T*_x_* film. Reproduced with permission.^[^ ^128^ ^]^ Copyright 2017, Nature Publishing Group. Cross‐sectional SEM images of f) 3D‐Ti_3_C_2_T*_x_*‐film and g) aerogel. h) The BET results of Ti_3_C_2_T*_x_*‐film, 3D‐Ti_3_C_2_T*_x_*‐film, and aerogel. Reproduced with permission.^[^ ^131^ ^]^ Copyright 2019, Elsevier.

MXenes, a new family of 2D transition metal carbides, carbonitrides, and nitrides, has been becoming a class of popular candidates for PC material since the first introduction of Ti_3_C_2_ by Gogotsi et al. in 2011, due to their integrated features of the high ion‐accessible surface area within the atomic‐scale layered structures, high *ρ* of ≈3.7 g cm^−3^, metallic electronic conductivity (≈10 000 S m^−1^), and good electrochemical stability.^[^ ^127^ ^]^ As shown in **Figure** **17**a, the unique structure of MXenes shows the following merits, which make them particularly attractive for high‐volumetric‐performance PCs: i) an excellent conductive inner transition metal carbide/carbonitride/nitride layer guarantees fast electron supply to electrochemically active sites; ii) a transition metal oxide‐like surface with high hydrophilicity is beneficial for the wettability of the electrode surface by electrolyte, facilitating the occurrence of pseudocapacitive redox reaction; iii) a 2D morphology and preintercalated water permit fast and efficient ion transport.^[^ ^128^ ^]^ Thus, tremendous research interests have been seen worldwide to apply this material into PCs.

Similar to graphene, the interlayer space in MXenes can be considered as continuous 2D nanochannels for electrolyte/ions transport; consequently, the dense stacking of these layers can severely reduce the accessibility to the electrolyte ions and raises the ion transport resistance, leading to unsatisfactory electrochemical utilization ratio. Recently, several strategies, such as expansion of the multilayers with large interlayer spacing and rational design of “porous/nanoporous and yet dense” architectures with high SSA and controlled pore features, have been widely developed for preventing self‐restacking of the MXene‐based film electrodes to boost their *C*_vol_. For example, the expansion of MXene multilayers can be realized by spontaneous intercalation of larger ions/hydrate ions (including Na^+^, K^+^, NH_4_^+^, Mg^2+^, Al^3+^) through electrochemical route,^[^ ^126^ ^]^ resulting in increased interlayer spacing. By comparison, the electrochemical intercalation of K^+^ into Ti_3_C_2_T*_x_* increased the *c*‐lattice parameter from 20.3 to 25.4 Å, and the binder‐free intercalated Ti_3_C_2_T*_x_* film electrode delivered a *C*_vol_ of 340 F cm^−3^ in aqueous electrolyte (Figure 17b). Similar interlayer expansion by ion interaction was also realized by other process, such as acid/alkali treatment.^[^ ^129^ ^]^ However, the restacking of the cation‐intercalated MXene flakes can still be fast and severe in the drying process, leading to compact MXene films that have limited electrolyte transport. To this end, Barsoum et al. reported that a vacuum filtration of the exfoliated Ti_3_C_2_T*_x_* suspension followed by rolling process was used to produce clay‐like additive‐free Ti_3_C_2_T*_x_* film with a high *C*_vol_ of 900 F cm^−3^ in aqueous electrolyte (Figure 17c,d), in which single‐layered Ti_3_C_2_T*_x_* flakes with large lateral size and good quality were present and interconnected.^[^ ^65g^ ^]^

On the side of rational design of the “porous and yet dense” architectures or to create proper interconnected nanochannels within MXene film electrode, the methods of process optimization (e.g., template‐assist and freeze drying) and addition of hetero species (e.g., CNT and graphene) as nanospacer have been developed to further improve their volumetric performance.^[^ ^128^, ^130^, ^131^, ^132^, ^133^, ^134^, ^135^, ^136^, ^137^, ^138^, ^139^, ^140^, ^141^, ^142^, ^143^, ^144^ ^]^ For instance, Gogotsi et al. used templating with polymethyl methacrylate (PMMA) microspheres to obtain macroporous MXene film electrodes with an open structure (Figure 17e), in which the ion transport lengths were greatly reduced, leading to an exceptional high‐rate performance and an acceptable volumetric performance.^[^ ^128^ ^]^ Freeze drying is a useful method to dry out the particular material while maintaining its looseness.^[^ ^131^, ^132^, ^133^ ^]^ The 3D macroporous film and aerogel with tightly interconnected layers were produced by liquid nitrogen rapid freezing (Figure 17f–h).^[^ ^131^ ^]^ Compared with the structure derived from normal vacuum drying, the 3D porous aerogel showed a higher SSA by 22 times and larger wedge‐shape pores with size of 1–2 µm, while maintaining a relatively large interlayer space of 1.1 nm. Consequently, the fabricated symmetric capacitor based on the porous MXene aerogel delivered a high *C*_vol_ of 1293 F cm^−3^ in aqueous electrolyte. More importantly, with an extra vacuum filtration before the rapid freeze, the obtained 3D MXene film would be more compact yet still highly porous. The film had narrower pore size distribution than that of aerogel, indicating a higher compactness; thus, a higher *C*_vol_ of 1355 F cm^−3^ and *E*_vol‐electrode_ of 32.2 Wh L^−1^ can be achieved for 3D MXene film‐based PC. Besides the extra vacuum filtration that brought in higher compactness, a post pressing or calendaring process to the freeze‐dried aerogel also achieved similar results.^[^ ^132^ ^]^ The post pressing or calendaring allows controllable thickness and compactness of the MXene aerogel by adjusting the applied force to balance the mass density and the porosity. From the calendaring method, its thickness was reduced homogenously while maintaining the integrity of lamellae structure and accessible surface area, leading to higher mass density. Optimally with a mass density of 1030 mg cm^−3^, a *C*_vol_ of 323 F cm^−3^ was achieved for MXene aerogel‐based PC.

**Figure 18:**
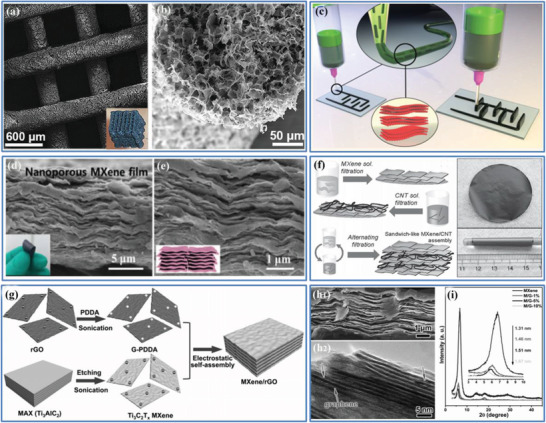
a,b) SEM image of freestanding and porous MXene microlattice. Reproduced with permission.^[^ ^135^ ^]^ Copyright 2019, Wiley‐VCH. c) Schematic drawing demonstrating the 3D printing of MCs with interdigital architectures. Reproduced with permission.^[^ ^136^ ^]^ Copyright 2020, American Chemical Society. d,e) Cross‐sectional SEM images of the nanoporous MXene film. Reproduced with permission.^[^ ^137^ ^]^ Copyright 2018, Royal Society of Chemistry. f) Schematic showing the preparation of the sandwich‐like CNT@MXene films and their digital photographs. Reproduced with permission.^[^ ^142^ ^]^ Copyright 2015, Wiley‐VCH. g) Schematic illustration for the synthesis of the rGO@MXene hybrids. h_1_) Cross‐sectional SEM of rGO@MXene hybrid film. h_2_) TEM images of rGO@MXene hybrids. i) XRD patterns of the prepared MXene and rGO@MXene hybrids. Reproduced with permission.^[^ ^143^ ^]^ Copyright 2017, Wiley‐VCH.

More recently, 3D printing technique, i.e., additive manufacturing (AM), has demonstrated great potential in development of self‐standing and hierarchically porous framework for EC materials and devices because it can directly produce complex 3D architecture.^[^ ^134^ ^]^ For example, utilizing the aqueous ink composed of atomically thin 2D Ti_3_C_2_T*_x_* with large lateral size of about 8 µm, the Ti_3_C_2_T*_x_* layers were assembled into freestanding and porous architecture with high SSA through 3D printing technique (**Figure** **18**a,b).^[^ ^135^ ^]^ The thus‐produced architecture had large pores for electrolyte infusion and surface wetting, while these Ti_3_C_2_T*_x_* thin layers (1–3 nm thickness) within the backbone were randomly interconnected into 3D porous structures with large continuous channels, providing full accessible surface to the electrolyte/ions. As a result, the 3D‐printed device delivered an acceptable volumetric performance and outstanding cycling stability. In another example, Beidaghi et al. reported that the fabrication of all‐solid‐state microcapacitors (MCs) through a 3D printing of additive‐free and water‐based MXene ink (Figure 18c).^[^ ^136^ ^]^ Benefiting from the high electrical conductivity and excellent electrochemical properties of 2D MXene flakes and the 3D interdigital electrode with a hierarchically porous architecture, the fabricated MC delivered a high *E*_vol‐electrode_ of 56 mWh cm^−3^ in aqueous electrolyte.

One of the other methods to design “porous and yet dense” MXene films is the addition of hetero species into the MXene layers. In detail, exfoliated Ti_3_C_2_T*_x_* suspension was first mixed with a hetero species suspension with a controlled mass ration. Second, the composite film with a 3D porous network was obtained by vacuum filtration of the mixture solution followed by drying and/or removal of the added hetero species. The hetero species, either 0D or 1D additives, act as spacer to separate and stabilize the MXene layers during drying process. Typical examples of spacers included metal oxides (e.g., Fe(OH)_3_ nanoparticles,^[^ ^137^ ^]^ MgO nanoparticles,^[^ ^138^ ^]^ and MnO_2_ nanowires^[^ ^140^ ^]^) and organic materials (e.g., polystyrene microspheres).^[^ ^139^ ^]^ It should be noted that the pore features in the produced 3D porous networks are strongly dependent on the spacer size/geometry and loading. In all cases, expanded interlayered structures that still densely packed were obtained, leading to enhanced volumetric performance over their nonporous counterparts. For example, a free‐standing and nanoporous MXene film was fabricated by incorporating Fe(OH)_3_ nanoparticles (a diameter of 3–5 nm) into MXene films and then dissolving the Fe(OH)_3_ nanoparticles, followed by low temperature calcination at 200 °C (Figure 18d,e), leading to highly interconnected nanopore channels that facilitate efficient and rapid transport electrolyte ions.^[^ ^137^ ^]^ Consequently, the modified film electrode delivered a high *C*_vol_ of 1142 F cm^−3^ in aqueous electrolyte; and even at a high mass loading of 11.2 mg cm^−2^, the *C*_vol_ can be maintained at 749 F cm^−3^. Similarly, when the MnO_2_ nanowires (a diameter of 20–30 nm and length of up to 50 µm) were inserted into the porous MnO_2_@MXene composite film, a *C*_vol_ of 1025 F cm^−3^ and an *E*_vol‐electrode_ of 56.94 mWh cm^−3^ were achieved for an all‐solid‐state symmetric capacitor based on MnO_2_@MXene films.^[^ ^140^ ^]^

Besides the above‐mentioned hetero species, carbon materials (e.g., CNT^[^ ^142^, ^144^ ^]^ and graphene^[^ ^141^, ^143^, ^145^ ^]^) with various nanostructures have also been introduced into MXenes to produce porous carbon@MXene composite films with improved volumetric performance. In this strategy, the carbon materials can not only be served as effective nanospacer to expand the nanofluidic channels of MXene multilayers, facilitating the reversible intercalation reactions of charge carriers, but also effectively prevents self‐restacking of MXene flakes. Moreover, they will further improve the volumetric capacitance of composite film electrode as the EDLC active materials. Two typical and most efficient candidates are 1D CNT and 2D graphene. For example, Gogotsi et al. have proposed a simple method for the fabrication of flexible, sandwich‐like CNT@MXene composite film electrodes by alternating filtration of MXene and CNT dispersions (Figure 18d).^[^ ^142^ ^]^ Compared with the pure MXene and randomly mixed CNT@MXene films, the sandwich‐like CNT@MXene one showed a more ordered and porous structure, which provided additional and faster diffusion paths for electrolyte ions. As a result, the sandwich‐like CNT@MXene film electrodes exhibited the highest *C*_vol_ of 390 F cm^−3^ and excellent rate performance among the three samples. In another work, utilizing biscrolling technique, a flexible yarn electrode containing predominantly MXene nanosheets (up to ≈98 wt%) that were trapped within CNT yarn scrolls (denoted as BXM yarns) was fabricated by Razal et al. An all‐solid‐state asymmetric fiber‐shaped EC based on the BXM yarns as negative electrode and biscrolled RuO_2_ yarns as positive electrode delivered a high *E*_vol‐electrode_ of 61.6 mWh cm^−3^.^[^ ^144^ ^]^ On the side of graphene spacer, by using electrostatic self‐assembly between positively charged rGO modified with poly(diallyldimethylammonium chloride) and negatively charged MXene nanosheets, a flexible and conductive rGO@MXene composite was obtained, in which rGO nanosheets are successfully inserted in‐between MXene layers (Figure 18h, 2), leading to a considerably increased interlayer spacing (Figure 18i).^[^ ^143^ ^]^ The optimal rGO@MXene composite film displayed a *C*_vol_ of 1040 F cm^−3^, an impressive rate capability, and long cycle life. Moreover, similar graphene@MXene composite film had also been prepared by vacuum‐assisted filtration method, which can deliver a higher volumetric performance than their pure MXene counterparts.^[^ ^141^ ^]^

Although the volumetric capacitance and energy density are not as impressive as what have been expected in some of above‐mentioned works, due to the loose packing of the layers (low *ρ*) and the wide pore size distribution in the 3D porous network, they have indeed provided meaningful insights on how to uniformly introduce CNT or graphene spacer into MXene layers to obtain composite film with improved electrochemical performance. In addition, what has to be noted for this high‐volumetric‐performance works is that the assembled CNT@MXene or graphene@MXene composite layers, although have well‐balanced pore features and *ρ*, are parallel to the current collector. This is expected to limit the ion transport in thick electrodes and thus makes the capacitance performance highly thickness dependent. Significantly, a vertically aligned MXene arrays on current collector were prepared via applying mechanical shearing force to high‐order lamellar MXene liquid crystals and strengthened by intercalated CNTs.^[^ ^146^ ^]^ In addition to the dense packing of MXene layers with interconnected nanochannels for the transport electrolyte ions, the vertical alignment of the layers allows for fast and directional transport of electrolyte, leading to thickness‐independent capacitance performance of up to 200 *μ*m.

## 5 Summary and Perspectives

ECs have been widely pursued as among the key energy storage devices, especially for those high‐power applications. However, their energy storage abilities (i.e., energy density) are obviously much lower than those of LIBs, making them far from being ideal the ever‐growing energy storage demands. The past three decades have witnessed tremendous research and development efforts devoted to improving the principal performance parameters of ECs, such as energy density, without sacrificing other merit parameters, such as the high‐power performance and long‐cycling stability. Although significant achievements have been made so far, regrettably, the volumetric performance was largely ignored/overlooked, while considerable attentions were placed to the gravimetric energy density. For many of the existing and arising applications, volumetric performance is a more relevant metric than the gravimetric one, especially for those applications where space limitations are among the concerns. Thus, it would be of great importance to design ECs that can store as much energy as possible within limited space. For electrodes, a critical requirement shall be appropriately “porous and yet dense,” in order to realize the high volumetric performance. For ECs, carbon‐based materials are among the best choices, together with other active materials, depending on the types of ECs.

As clarified in this review, volumetric performance is a crucial technical parameter in the future design and development of ECs. It can be achieved through the rational design and development of highly “porous and yet dense” electrodes, as has been examined on the basis of porous carbon‐based electrode. There have been a number of recent studies in tuning the structures of these porous electrode materials, mainly via the various pore‐engineering approaches, and leading to a rise in volumetric performance. Nonetheless, there are several challenges that need to be addressed.

The first and key challenge in designing and developing a class of highly “porous and yet dense” electrodes with high volumetric performance is to balance the trade‐offs between *ρ* and pore features (such as the pore volume, pore size, pore size distribution, pore connectivity, and pore wettability) of different EC materials. In recent years, several new strategies of pore engineering in improving the volumetric performance of porous electrode have been reported. Taking engineering of pore sizes in carbon‐based materials as an example, tuning the type and distribution of pores, nurturing surface area, incorporating appropriate spacers to enlarge micropores can enhance the overall volumetric performance. They effectively increase the *ρ*, keep a large ion‐accessible surface area and unblock transport channels for the efficient diffusion of electrolyte ions. However, it should be noted that the volumetric performance of these carbon‐based materials is also limited to a certain extent from their intrinsically low double‐layer capacitance and thus energy density. To this end, the incorporation of appropriate pseudocapacitive materials (such as transition metal oxides/hydroxides and conductive polymers) into carbon materials of different dimensions, whereby making multicomponent nanocomposites, can further increase the overall volumetric performance. They take the advantage of combining the individual structural components and the synergistic effects among the individual components. However, care shall be taken such that the rate capability and cycling stability shall not be significantly affected, as one has to consider the inherently poor electronic conductivity and obvious volume changes in association with the redox‐active pseudocapacitance materials during the charging/discharging processes. Therefore, optimizing the interfacial interactions among the pseudocapacitive components and carbon‐based materials and designing an optimum nanocomposite structure shall be considered.

Second, to fulfill the technical demand of ECs being used in large scales, it would be of great importance to well keep the desirable volumetric performance, when the mass loading of porous electrode is raised to the commercial level (e.g., 10 mg cm^−2^). Along this line, the key pore features, such as pore volume, size distribution, pore connectivity, and pore tortuosity, in the high‐mass‐loaded or thick film electrodes (>10 mg cm^−2^ or 100–200 µm) should be properly controlled, where porous electrode structures with a continuous conductive network for electron transport and a straight and aligned channel (pore) for fast ion transport are required, when compared with the low‐mass‐loaded counterparts or thin films (0.5–1 mg cm^−2^ or 5–15 µm). The mass transport limit of electrolyte ions will become increasingly significant in these high‐mass‐loaded or thick film electrodes. In this connection, it would be necessary to re‐examine the fundamental design principles of pore features more carefully, thus reducing the resistances of ion transport and charge transfer without compromising the high‐mass‐loading requirement. To realize this target, the following considerations would be useful in aiming for a commercial‐level mass loading of electrodes for ECs: i) optimizing the chemical properties, e.g., by heteroatoms/ions in carbon‐based materials; ii) configuring a hierarchical pore structure with a straight and aligned channel (pore); iii) creating more appropriate nanopore channels; iv) combining with an appropriate 3D conductive substrate/current collect; and v) aligning an appropriate internal construction by physical force/field.

Third, although considerable advances have been made with the fundamental understanding on the charge‐storage mechanisms of ion transport and adsorption of “sub‐nanoporous and ultra‐dense” electrodes, parts of the full picture still remain rather unclear/incomplete, especially when a set of more complex pore features/structures is taken into consideration, and/or with a redox reaction of pseudocapacitive materials being involved. Thus, it would be imperative to develop and employ more advanced in situ or operando techniques (such as in situ dissipative EQCM, NMR spectroscopy, SAXS, X‐ray diffraction (XRD), and electrochemical atomic force microscopy) to gain more in situ information on the electrode/electrolyte interface interaction. In addition, when coupled with some of these advanced characterization techniques, simulation and modeling (such as molecular dynamics simulation and DFT approach) would be another set of effective tools for digging out extra information, such as on the ion distribution and population, thus analyzing/understanding the underlying theory and predicting optimized pore features/structures within the porous and yet porous carbon materials.

Fourth, as both the energy density and power density of ECs are proportional to the square of the voltage window, there shall be more pay‐off by broadening the working voltage of ECs through selection of an appropriate electrolyte that allow for a large voltage window (up to 2.5–2.8 V). Note that the IL electrolytes are not suitable to fulfill the practical demand of ECs, due to their high cost. Thus, controls in the well‐matching between the pore size in carbon‐based electrodes and the organic electrolyte ions become fairly important in achieving an overall high‐volumetric performance. In other words, a nanoporous structure in electrode materials shall be properly designed, in order to realize an ultrahigh volumetric capacitance with the selected electrolyte. Recent research has identified that the partial removal or distortion of the ion solvation shell shall be considered, in order to make a good match when the pore size is close to the bare ion size.

Finally, in connection with the powder‐form materials, it should be pointed out that their ESSA, *ρ*, and pore features/structures can be largely affected by the fabrication procedure and processing parameters. This also applies to those active materials applied in an electrode, where there can be additional binders (and conductive additives if necessary), especially for those high‐mass‐loaded or thick film electrodes (>10 mg cm^−2^ or 100–200 µm). One of the useful approaches in engineering the relationships between the porous active material and binders (and conductive additives if necessary) is to keep a well‐balance with the level of porosity and *ρ*. For example, compressing a porous electrode at too high pressure can collapse part of the pores and decrease the conductive paths of electrolyte ions.

Besides the above‐mentioned challenges restricting to development of “porous and yet dense” electrodes for high volumetric performance, it would also be essential to look at several technical issues. For example, noted that it is important to precisely calculate the thickness of a porous electrode than that of the electrode weight, leading to an obvious calculation/estimation error for volumetric performance. In addition, there is need for a degree of unification in the characterization techniques and methods for electrochemical performance, as there is a current‐faced lack of commonly standardized and accepted guidelines/criteria, such as various electrochemical working stations, measurement of electrochemical performance of electrodes and devices, and calculation methods of *ρ* and *C*_vol_. They could result in huge data error and be misleading to a precise comparison of the performance. Thus, there is need for establishing a set of international standards for electrochemical tests and performance comparison of electrodes and devices.

For the past three decades, ECs as a key type of energy storage devices have been developed extensively, and numerous progresses have bene made in the electrode materials and devices. The continued maturation of the existing ECs and arising of the new prototypes are dependent on the design and construction of porous architectures and configurations, where for high‐volumetric performance, there shall be a balance of several “porous and yet dense” structure parameters. They are also applied to other types of energy storage devices, such as batteries and hybrid‐types.

## Conflict of Interest

The authors declare no conflict of interest.
